# Supplementary material for: TMED2 binding restricts SMO to the ER and Golgi compartments
Source: PLoS Biol. 2022 Mar 30;20(3):e3001596. doi: 10.1371/journal.pbio.3001596 (PMC9000059; doi:10.1371/journal.pbio.3001596)
Supplement: S1 Table — Top 10 candidates identified in PMP screening and sorted according to HaSAPPy score. Number of I.I. and D.I. detected in control and selected samples are provided for each gene. Subcellular localization of corresponding proteins is indicated (Cell. comp.). D.I., disrupting insertion; I.I., independent insertion; PMP, purmorphamine. (PDF) [file pbio.3001596.s001.pdf]

S1 Table I Selected candidate gene list

Candidate	Score	I.I.		D.I.		Cell. comp.	Reference
		Control	Selected	Control	Selected		
Myh9	27.8	98	353	48	252	Cytoskeleton	(Takubo et al. 2003)
Tmed10	25.1	84	251	58	238	ER-Golgi	(Strating and Martens 2009)
Tmed2	21.6	18	49	11	46	ER-Golgi	(Strating and Martens 2009)
Tmem41b	19.8	24	66	16	57	ER	(Moretti et al. 2018)
Trim71	17.6	74	130	41	117	Cytoplasm	(Chang et al. 2012)
Pitpnb	17.0	153	399	87	251	Golgi	(Phillips et al. 2006)
Vmp1	16.6	194	365	111	322	ER	(Tabara et al. 2018)
Rab14	14.2	104	190	52	139	Golgi	(Lindsay and McCaffrey 2015)
Sec63	*14.2*	*280*	*528*	*186*	*464*	*ER*	*(Ast et al. 2013)*
Tpm3	*10.8*	*31*	*51*	*14*	*37*	*Cytoskeleton*	*(Bryce et al. 2003)*
