# Supplementary material for: TMED2 binding restricts SMO to the ER and Golgi compartments
Source: PLoS Biol. 2022 Mar 30;20(3):e3001596. doi: 10.1371/journal.pbio.3001596 (PMC9000059; doi:10.1371/journal.pbio.3001596)
Supplement: S2 Table — Six neural tube sections of E9.0 WT and E10.5 Tmed2−/− embryos were analyzed and cells positive for the OLIG2 and NKX6.1 were counted. Percentage of OLIG2 relative to NKX6.1 positive cells is provided for each section. WT, wild-type. (PDF) [file pbio.3001596.s002.pdf]

S2 Table I OLIG2 and NKX6.1 positive cells in neural tube sections of *Tmed2*$^{-/-}$ and WT embryos

WT E9.0

**Section**	1	2	3	4	5	6
**OLIG2+/NKX6.1+**	10	15	14	47	37	43
**NKX6.1 +**	70	115	119	144	121	134
**Ratio**	14%	13%	11%	32%	30%	32%

WT E10.5

**Section**	1	2	3
**OLIG2+/NKX6.1+**	101	107	45
**NKX6.1 +**	468	752	395
**Ratio**	21%	14%	11%

***Tmed2*$^{-/-}$ E10.5**

**Section**	1	2	3	4	5	6
**OLIG2 +/NKX6.1+**	33	29	42	39	30	32
**NKX6.1 +**	62	59	74	75	66	68
**Ratio**	53%	49%	57%	52%	45%	47%
