# Supplementary material for: TMED2 binding restricts SMO to the ER and Golgi compartments
Source: PLoS Biol. 2022 Mar 30;20(3):e3001596. doi: 10.1371/journal.pbio.3001596 (PMC9000059; doi:10.1371/journal.pbio.3001596)
Supplement: S1 Text — (PDF) [file pbio.3001596.s003.pdf]

Supplemental References

Ast T, Cohen G, Schuldiner M. 2013. A network of cytosolic factors targets SRP-independent proteins to the endoplasmic reticulum. *Cell* **152**: 1134-1145.

Bryce NS, Schevzov G, Ferguson V, Percival JM, Lin JJ, Matsumura F, Bamburg JR, Jeffrey PL, Hardeman EC, Gunning P et al. 2003. Specification of actin filament function and molecular composition by tropomyosin isoforms. *Mol Biol Cell* **14**: 1002-1016.

Chang HM, Martinez NJ, Thornton JE, Hagan JP, Nguyen KD, Gregory RI. 2012. Trim71 cooperates with microRNAs to repress Cdkn1a expression and promote embryonic stem cell proliferation. *Nat Commun* **3**: 923.

Chen G, Hou Z, Gulbranson DR, Thomson JA. 2010. Actin-myosin contractility is responsible for the reduced viability of dissociated human embryonic stem cells. *Cell Stem Cell* **7**: 240-248.

Desouza-Armstrong M, Gunning PW, Stehn JR. 2017. Tumor suppressor tropomyosin Tpm2.1 regulates sensitivity to apoptosis beyond anoikis characterized by changes in the levels of intrinsic apoptosis proteins. *Cytoskeleton (Hoboken)* **74**: 233-248.

Haystead TA. 2005. ZIP kinase, a key regulator of myosin protein phosphatase 1. *Cell Signal* **17**: 1313-1322.

Joo EE, Yamada KM. 2014. MYPT1 regulates contractility and microtubule acetylation to modulate integrin adhesions and matrix assembly. *Nat Commun* **5**: 3510.

Lindsay AJ, McCaffrey MW. 2015. Rab antibody characterization: comparison of Rab14 antibodies. *Methods Mol Biol* **1298**: 161-171.

Moretti F, Bergman P, Dodgson S, Marcellin D, Claerr I, Goodwin JM, DeJesus R, Kang Z, Antczak C, Begue D et al. 2018. TMEM41B is a novel regulator of autophagy and lipid mobilization. *EMBO Rep* **19**.

Phillips SE, Ile KE, Boukhelifa M, Huijbregts RP, Bankaitis VA. 2006. Specific and nonspecific membrane-binding determinants cooperate in targeting phosphatidylinositol transfer protein beta-isoform to the mammalian trans-Golgi network. *Mol Biol Cell* **17**: 2498-2512.

Shi J, Wu X, Surma M, Vemula S, Zhang L, Yang Y, Kapur R, Wei L. 2013. Distinct roles for ROCK1 and ROCK2 in the regulation of cell detachment. *Cell Death Dis* **4**: e483.

Solimini NL, Liang AC, Xu C, Pavlova NN, Xu Q, Davoli T, Li MZ, Wong KK, Elledge SJ. 2013. STOP gene Phactr4 is a tumor suppressor. *Proc Natl Acad Sci U S A* **110**: E407-414.

Strating JR, Martens GJ. 2009. The p24 family and selective transport processes at the ER-Golgi interface. *Biol Cell* **101**: 495-509.

Tabara LC, Vicente JJ, Biazik J, Eskelinen EL, Vincent O, Escalante R. 2018. Vacuole membrane protein 1 marks endoplasmic reticulum subdomains enriched in phospholipid synthesizing enzymes and is required for phosphoinositide distribution. *Traffic* **19**: 624-638.

Takubo T, Wakui S, Daigo K, Kurokata K, Ohashi T, Katayama K, Hino M. 2003. Expression of non-muscle type myosin heavy polypeptide 9 (MYH9) in mammalian cells. *Eur J Histochem* **47**: 345-352.

Walker A, Su H, Conti MA, Harb N, Adelstein RS, Sato N. 2010. Non-muscle myosin II regulates survival threshold of pluripotent stem cells. *Nat Commun* **1**: 71.
