# Supplementary figures and images for: TMED2 binding restricts SMO to the ER and Golgi compartments
Source: PLoS Biol. 2022 Mar 30;20(3):e3001596. doi: 10.1371/journal.pbio.3001596 (PMC9000059; doi:10.1371/journal.pbio.3001596)

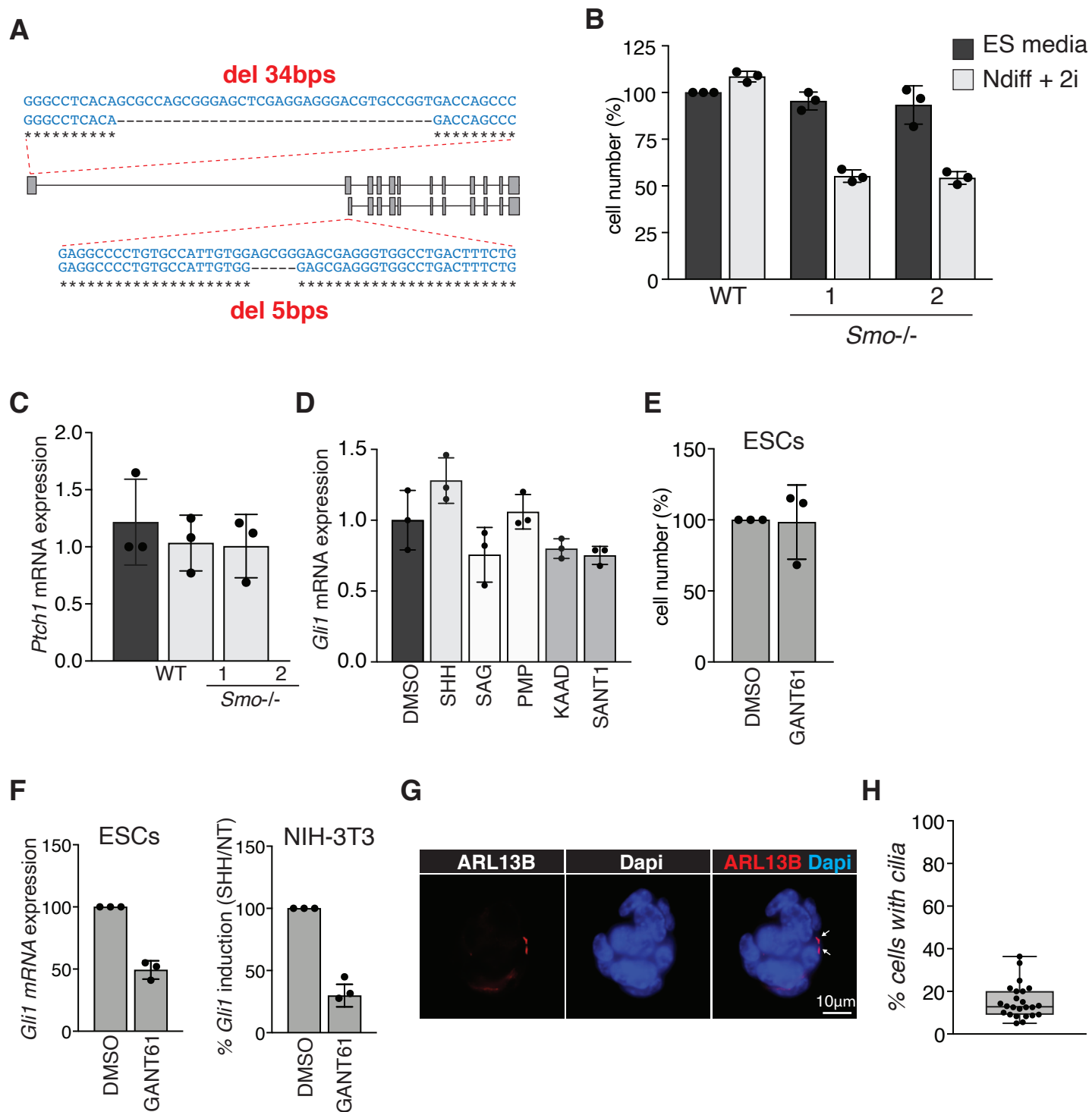

Supplement: S1 Fig — (A) Generation of Smo−/− ESCs by CRISPR/Cas-9 gene editing. Scheme showing Smo gene structure and transcripts variants. gRNAs were designed against the first exon of both transcripts. Sequence of the locus is shown for WT cells and for 2 clones where Cas-9–dependent NHEJ repair introduced frameshift mutations in the Smo coding region. Both mutations lead to a frameshift in the Smo coding sequence compromising protein expression. (B) Proliferation of WT and Smo−/− ESCs in ES and Ndiff+2i media. (C) GLI transcription is not affected by Smo deletion in ESCs. Ptch1 mRNA levels were measured by qPCR (n = 3, biological replicates). (D) GLI transcription remains unchanged in ESCs treated with HH pathway targeting compounds. ESCs were treated for 48 hours with SΗΗ (100 ng/ml), SAG (0.5 μM), PMP (1 μM), Cyclopamine-KAAD (1 μM), and SANT-1 (10 μM) as indicated, and Gli1 mRNA levels were measured by qPCR. Samples do not show statistically significant differences (n = 3, biological replicates). (E, F) Chemical inhibition of GLI activity does not affect ESCs survival. (E) Survival of ESCs after 48 hours with or without addition of the GLI inhibitor GANT61 (1μM). (F) Effect of GANT61 treatment on Gli1 expression. Expression of Gli mRNA with or without GANT61 (1μM) treatment in ESCs (n = 3, biological replicates) (left). Induction of Gli mRNA by SHH with or without GANT61 (1 μM) treatment in NIH-3T3 (n = 3, biological replicates) (right). (G, H) Frequency of ciliated ESCs. (G) Immunofluorescence showing ciliated cells in an ESC colony. ARL13B marker was used to stain cilia (indicated with an arrow). Scale bar = 10 μm. (H) Plot of the percentage of ciliated cells over the total number of cells. Each dot represents the percentage of cells with cilia detected in an ESC colony (n = 25). The data underlying all the graphs shown in the figure are included in the S1 Data file. ESC, embryonic stem cell; gRNA, guide RNA; HH, hedgehog; NHEJ, nonhomologous end joining; PMP, purmorphamine; qP [file pbio.3001596.s004.pdf]

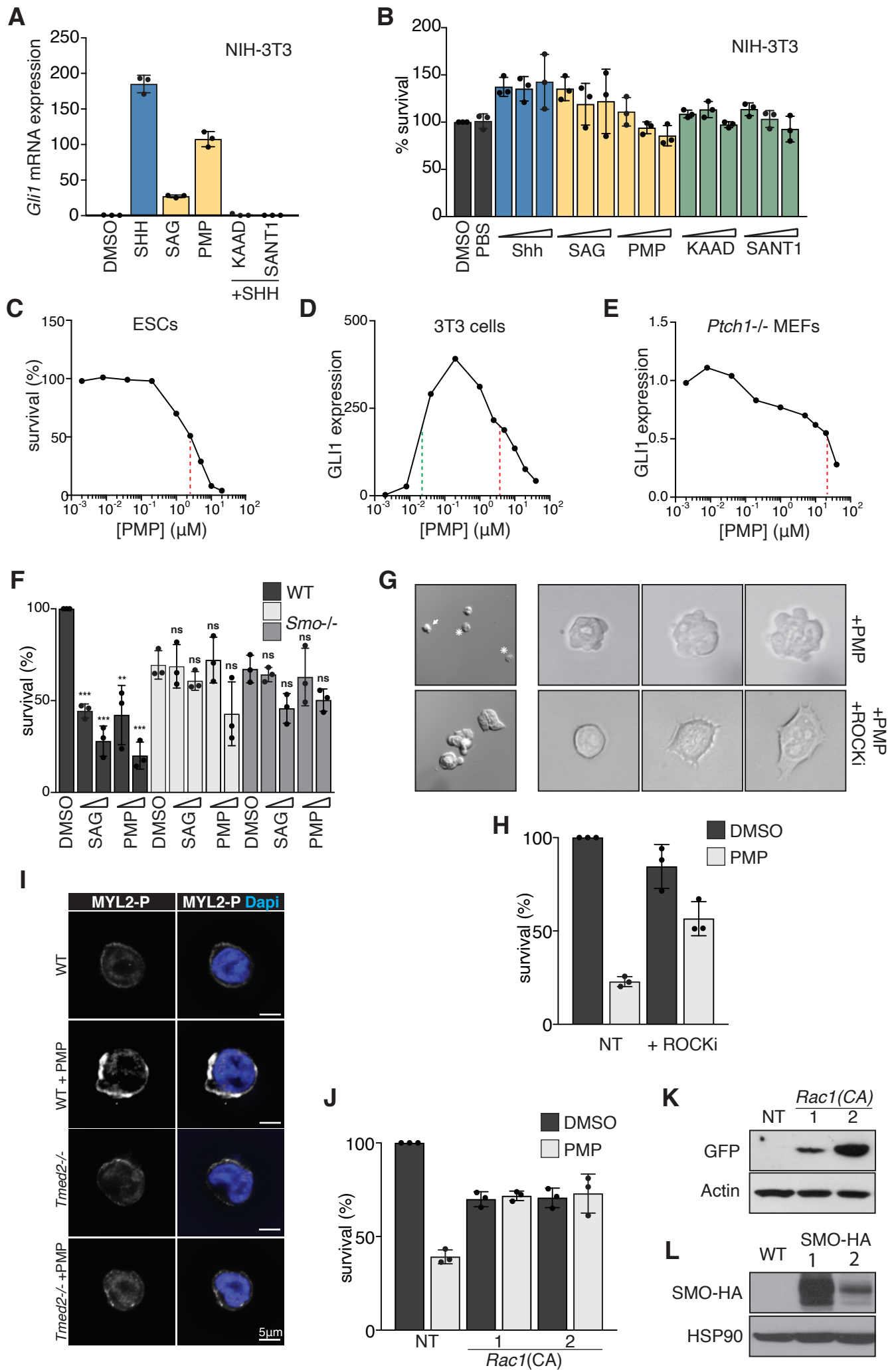

Supplement: S2 Fig — (A) Gli transcriptional activity in NIH-3T3 cells treated with compounds shown in Fig 2B. Gli1 mRNA was measured by RT-qPCR. (B) Effects of compounds indicated on survival of NIH-3T3 cells. NIH-3T3 cells were treated for 48 hours with SΗΗ (50–500 ng/ml), SAG (1–5 μM), PMP (2.5–10 μM), Cyclopamine-KAAD (1–5 μM) or SANT-1 (10–50 μM). Survival rate is normalized to DMSO treated sample. (C) Dissociation-induced apoptosis is prompted by high PMP concentrations. Survival of ESCs treated for 48 hours with different PMP concentrations (2 nM—20 μM). Red dotted line highlights IC50 at 2.5 μM. (D, E) High concentrations of PMP repress GLI activity and HH signaling. (D) RT-qPCR of Gli1 mRNA in NIH-3T3 cells treated with increasing concentrations of PMP (2 nM to 40 μM) and normalized to untreated samples. Dotted lines mark PMP EC50 (green, at 30 nM) and IC50 (red, at 4 μM). (E) RT-qPCR of Gli1 mRNA in Ptch1−/− MEFs treated with increasing concentrations of PMP (2 nM to 40 μM) and normalized to untreated samples. Red dotted line highlight IC50. (F) SAG and PMP cytotoxic effects are reduced in Smo−/− ESCs. Survival of Smo−/− and control ESCs treated for 48 hours with SAG (2.5 to 5 μM) and PMP (5 to 10 μM). The effect on 2 independent clones is shown. Asterisks denote statistical significance for difference from the DMSO treated samples. (G) PMP induces blebbing and death after dissociation of ESCs. On the left, ESC morphology 24 hours after dissociation and plating on Matrigel (upper panels, pretreated with PMP for 24 hours; lower panels, pretreated with PMP and with ROCKi). Cells showing membrane blebbing (arrow), and apoptotic bodies (asterisk) are indicated. Images at 1, 3, and 6 hours after plating are shown on the right. (H) Survival of ESCs treated for 48 hours with PMP with or without the addition of ROCKi. (I) Phosphorylated MYL2 (MYL2-P) distribution in WT and Tmed2 mutant ESCs treated with or without PMP for 24 hours. Bars represent 5 μm. (J) Constitutive active Rac1(CA) [file pbio.3001596.s005.pdf]

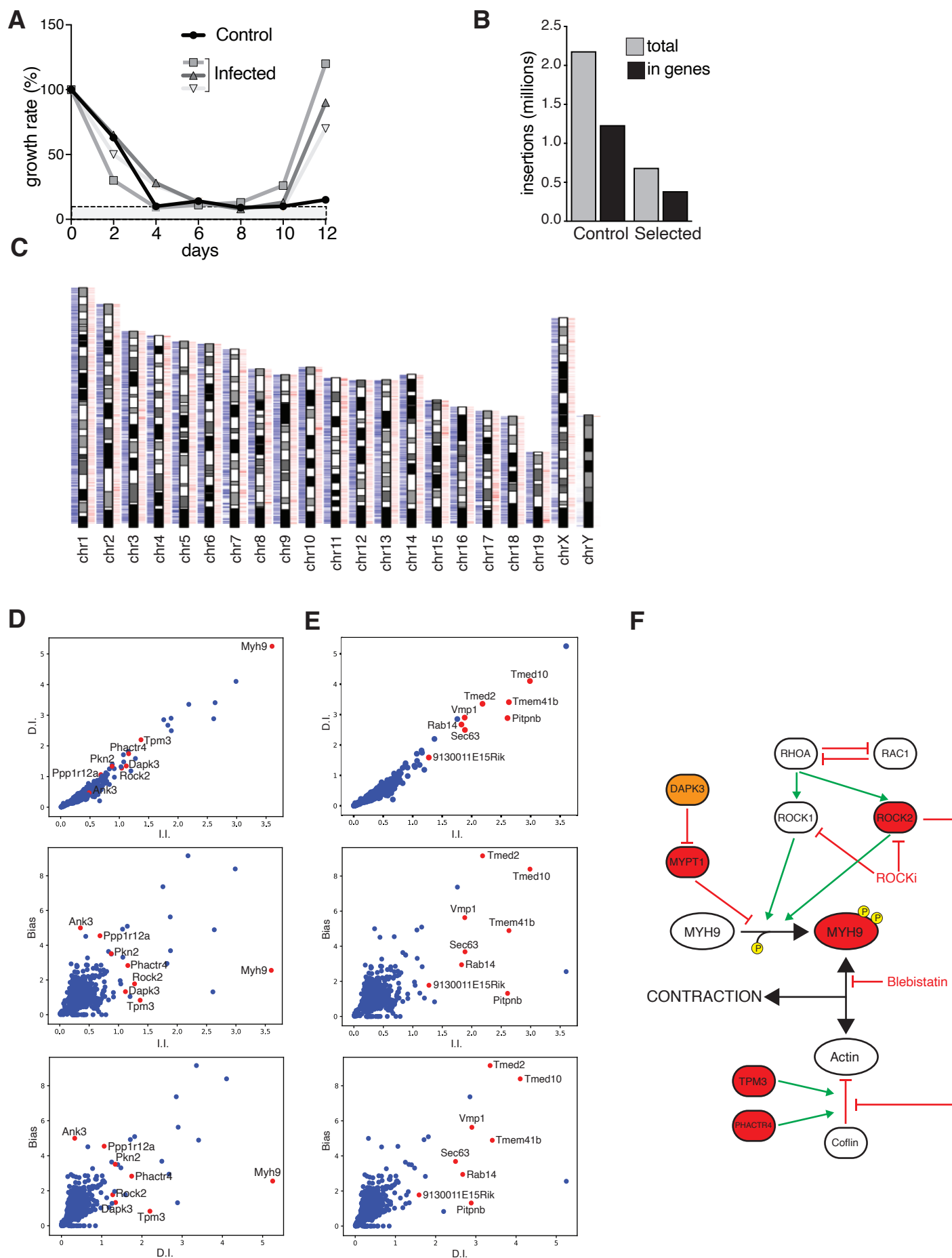

Supplement: S3 Fig — (A) Selection of ESCs resistant to PMP. Infected (3 independent experiments) and control cells were treated for 12 days with PMP. ESCs were passed and counted every 2 days. The graph shows the percentage of the counted cells relative to the number of cells plated on day 0. The dashed line indicates a baseline of irradiated MEFs used for ESC culture. (B) Depiction of insertion numbers genome wide (gray) and in gene transcription units (black), for control and PMP selected samples. (C) Chromosomal distribution of insertions in control (blue, above) and selected (red, below) samples are shown. (D, E) Two-dimensional plots of fold enrichment of I.I. and D.I. (left panel), I.I. and Bias (central panel), and D.I. and Bias (right panel) of genes during selection. Top hits genes involved in anchorage independent growth (D) or related to the ER–Golgi compartments (E) are marked in red and annotated. (F) Summary model showing the role of selected candidates in the Anoikis cascade: Myh9 [41,84], Phactr4 [85], Tpm3 [86], Rock2 [87], Mypt1 [88,89], and Dapk3 [89]. The data underlying all the graphs shown in the figure are included in the S1 Data file. ER, endoplasmic reticulum; ESC, embryonic stem cell; I.I., independent insertion; MEF, mouse embryonic fibroblast; PMP, purmorphamine. (PDF) [file pbio.3001596.s006.pdf]

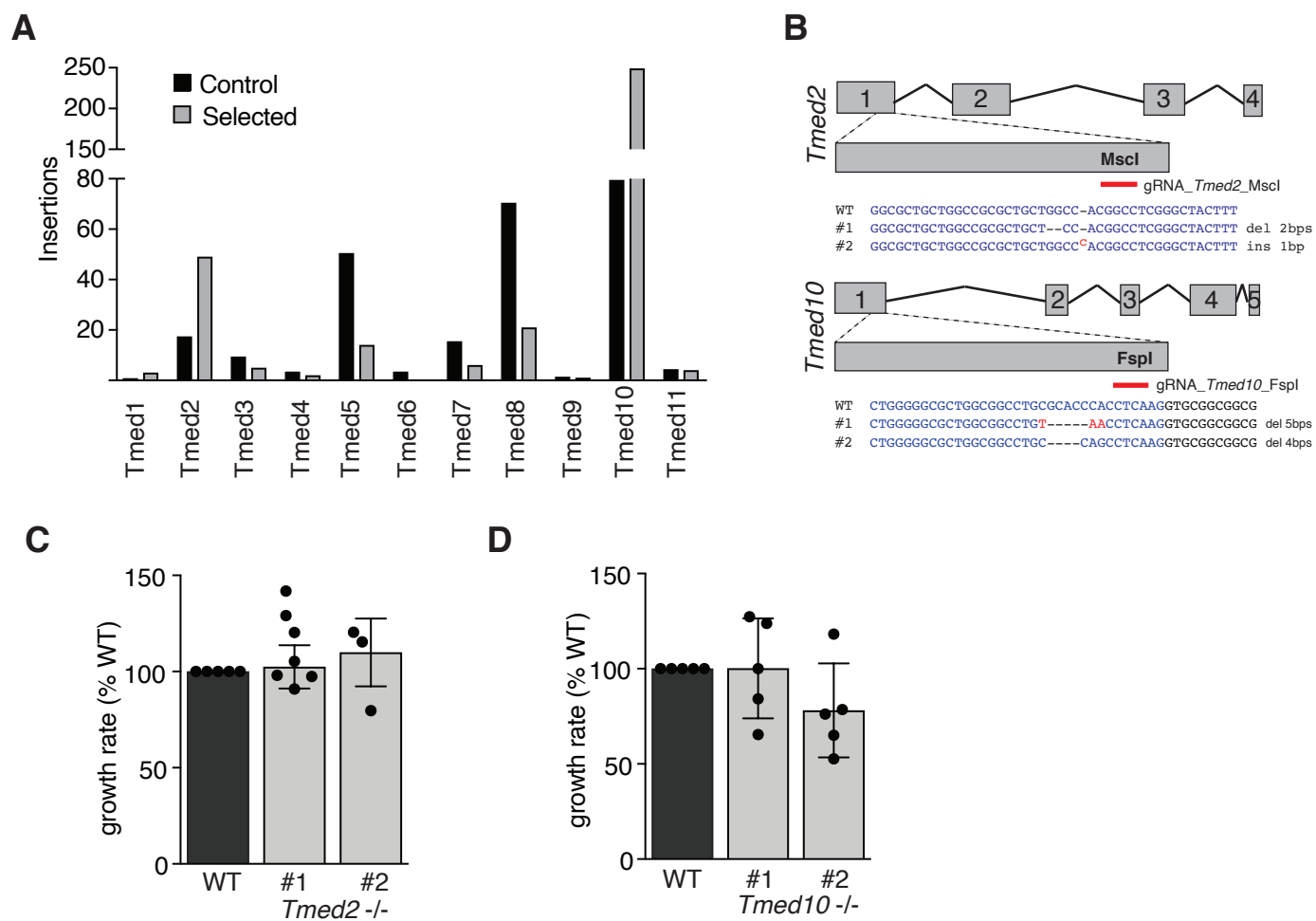

Supplement: S4 Fig — (A) Number of insertions in genes of the p24 family that were detected in selected and control samples. (B) Generation of Tmed2−/− and Tmed10−/− ESCs lines using CRISPR/Cas-9 technology. On the top schematic representation of the Tmed2 (above) and Tmed10 (below) gene locus with exons (numbers within boxes), gRNA position (red), and MscI (Tmed2) and FspI (Tmed10) restriction sites that were used to identify the gene edited clones are indicated. Sequence of the locus is shown for WT cells and for 2 clones where Cas-9 dependent NHEJ repair introduced frameshift mutations in the Tmed2 and Tmed10 coding region. Blue and black colors mark nucleotides in exonic and intronic regions, respectively. (C, D) Tmed2 and Tmed10 mutations are compatible with ESC self-renewal. Plots show relative growth rates of 2 independent Tmed2−/− and Tmed10−/− ESC clones compared to parental ESCs. Dots show individual measurements, error bars represent standard deviation (WT; n = 5). The data underlying all the graphs shown in the figure are included in the S1 Data file. ESC, embryonic stem cell; gRNA, guide RNA; HH, hedgehog; NHEJ, nonhomologous end joining; WT, wild-type. (PDF) [file pbio.3001596.s007.pdf]

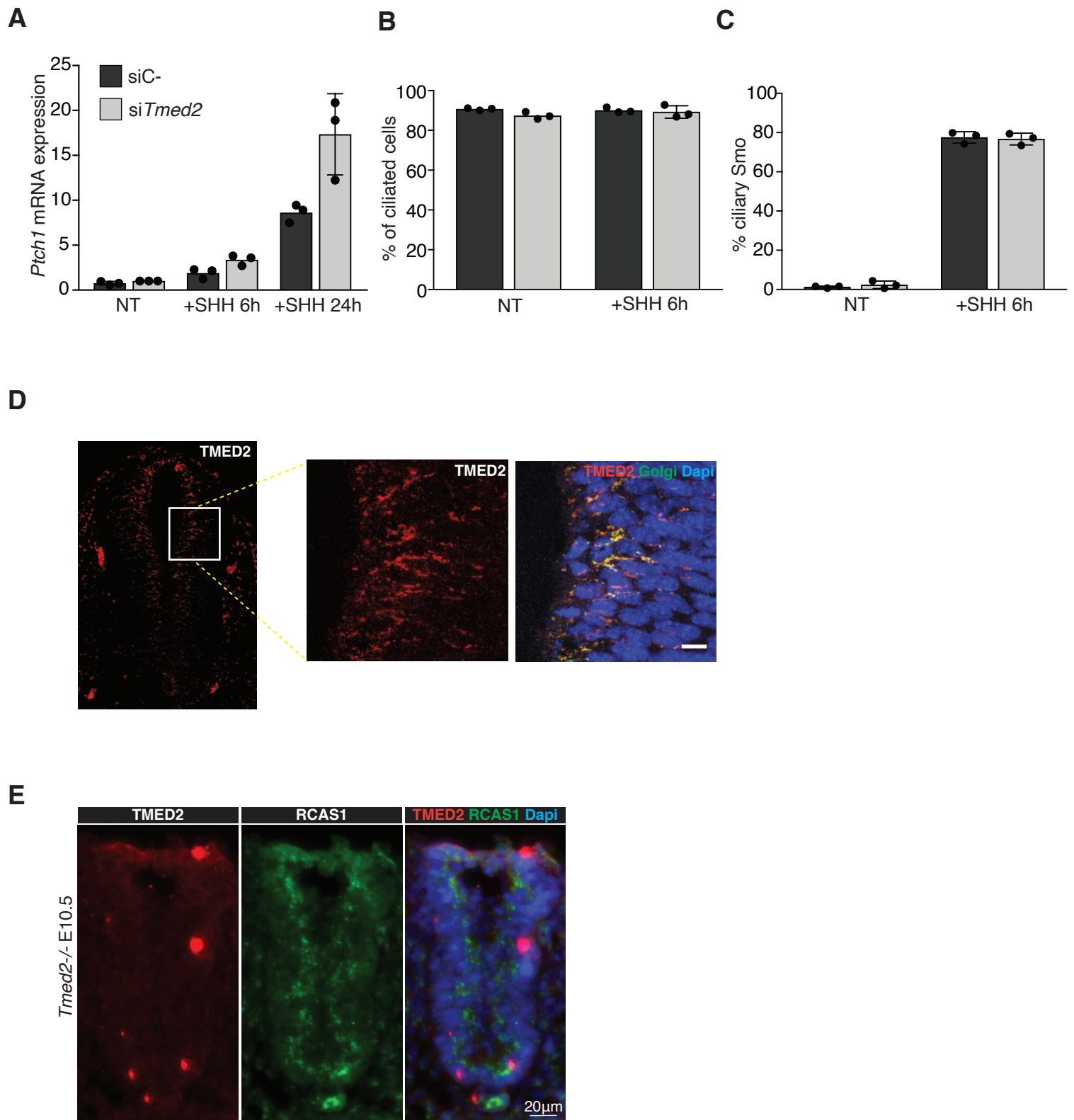

Supplement: S5 Fig — (A–C) Tmed2 depletion increases GLI transcriptional activity in NIH-3T3 cells. NIH-3T3 cells were transfected with either a siRNA targeting Tmed2 (siTmed2) or with a negative control siRNA (siC-) and treated with SHH (100ng/ml) for 6 or 24 hours. (A) RT-qPCR analysis of Ptch1 transcription; data are expressed as fold increase relative to siC-transfected cells. (B) Plot of the percentage of ciliated cells over the total number of cells. By immunofluorescence imaging, ARL13B marker was used to stain cilia in cells that were depleted for Tmed2 (black bars) or not (gray bars) as indicated. (C) Quantification of SMO recruitment to the primary cilia after 6 hours of SHH treatment in cells that were depleted for Tmed2 or not as in panel D. Number of cells positive for the SMO staining in primary cilia was normalized to the total number of counted cells. (D) TMED2 expression in the neural tube. Representative image of immunostainings of TMED2 and RCAS1 used to visualize the Golgi apparatus in neural tube sections of E9.5 embryos. Square indicates area magnified on the right. Scale bar = 10 μm. (E) Immunostaining of TMED2 (red) and the Golgi marker RCAS1 (green) in neural tube sections of Tmed2−/− E10.5 mouse embryos. Scale bar = 20 μm. The data underlying all the graphs shown in the figure are included in the S1 Data file. HH, hedgehog; RT-qPCR, quantitative reverse transcription PCR; SHH, Sonic hedgehog; siRNA, small interfering RNA; SMO, Smoothened. (PDF) [file pbio.3001596.s008.pdf]

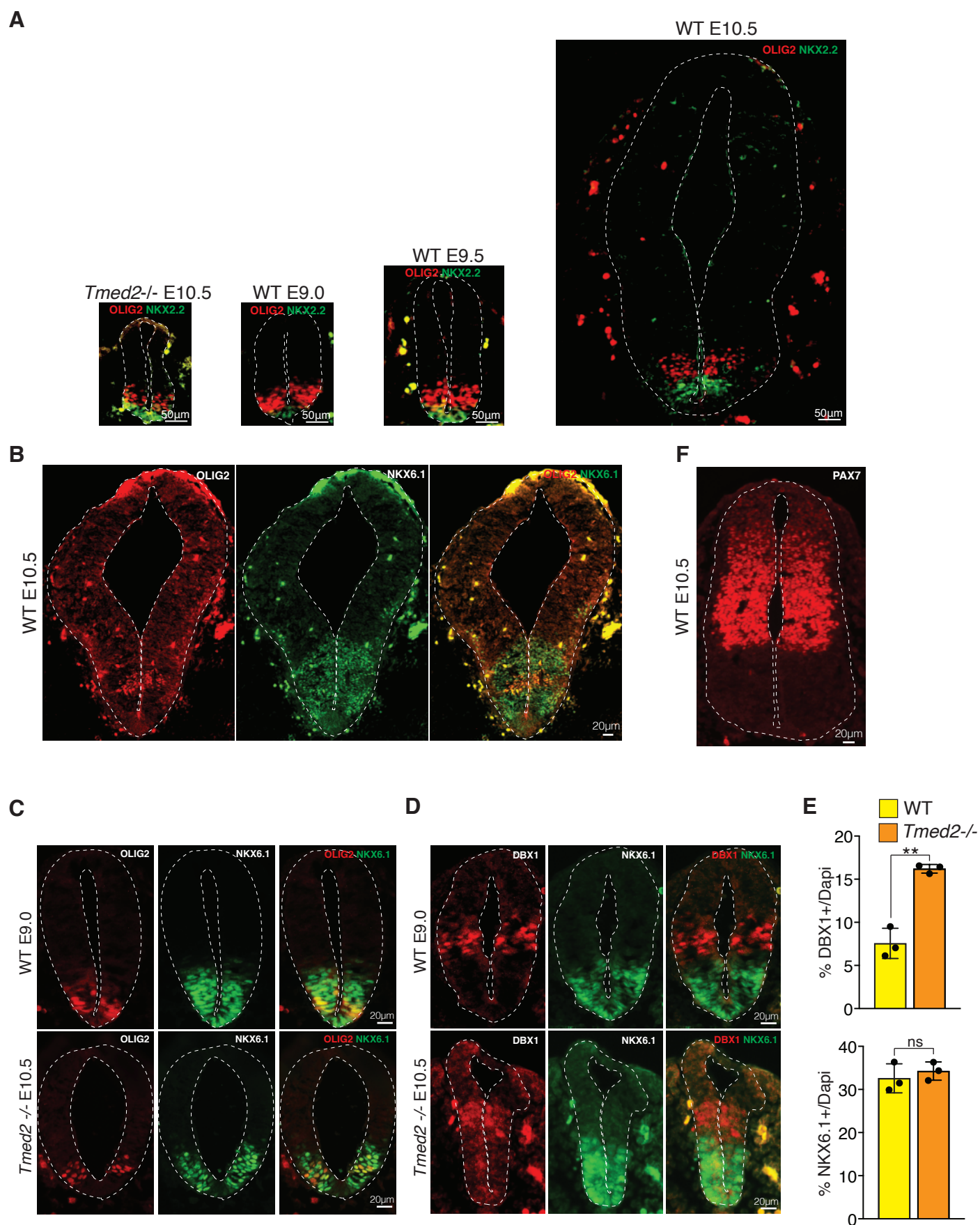

Supplement: S6 Fig — (A) Neural tube sections of E10.5 Tmed2−/−, E9.0, E9.5, and E10.5 control embryos stained for the ventral markers OLIG2 and NKX2.2. Scale bar = 20 μm. (B, C) Neural tube sections of E10.5 (B) and E9.0 control and E10.5 Tmed2−/− (C) embryos were stained for the ventral markers OLIG2 and NKX6.1. Scale bar = 20 μm. (D) Neural tube sections of E9.0 control and E10.5 Tmed2−/− embryos were stained for the markers DBX1 and NKX6.1. Scale bar = 20 μm. (E) Percentage of DBX1 (upper) and NKX6.1 (lower) expressing NPCs relative to total NPCs in neural tube sections of E9.0 control and E10.5 Tmed2−/− embryos. Asterisks denote statistical significance for difference between indicated samples. (F) PAX7 expression in a neural tube section of an E10.5 control embryo. Scale bar = 20 μm. The data underlying all the graphs shown in the figure are included in the S1 Data file. NPC, neural progenitor cell. (PDF) [file pbio.3001596.s009.pdf]

A

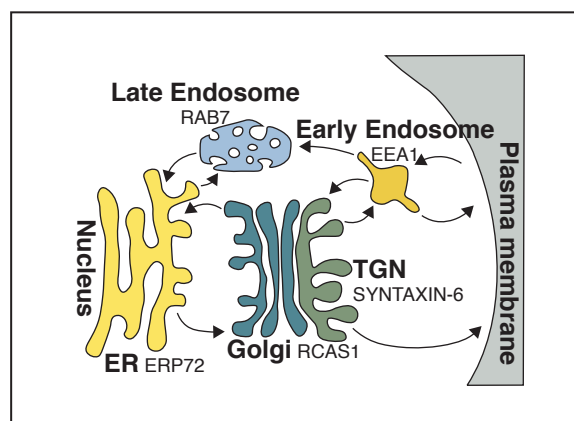

B

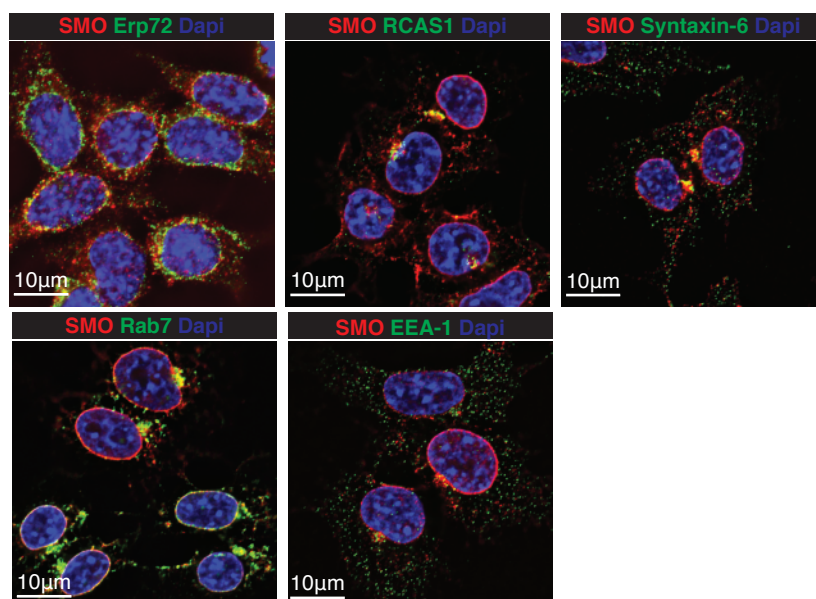

C

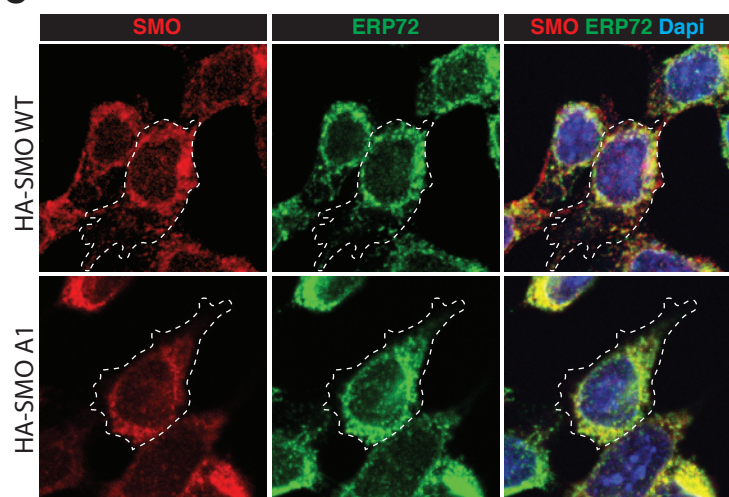

D

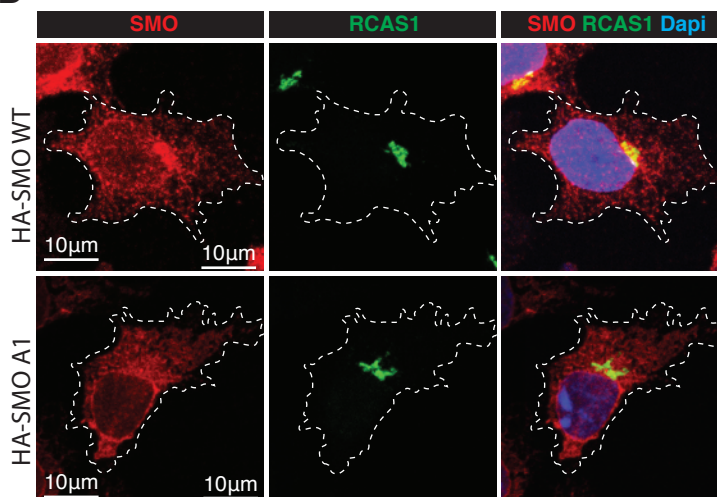

E

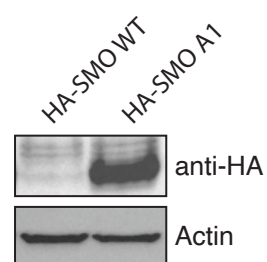

F

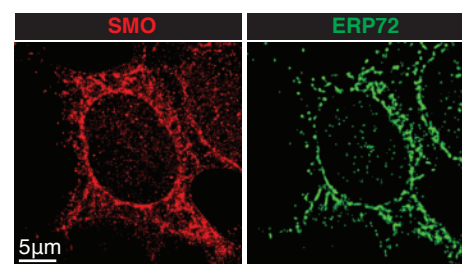

G

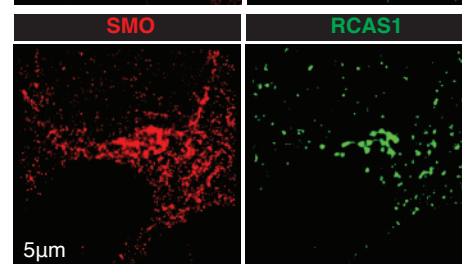

H

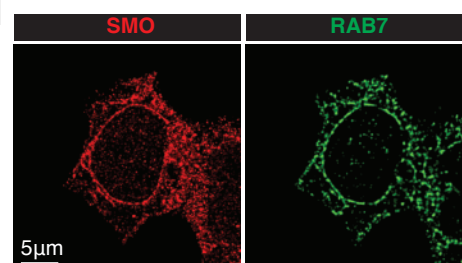

I

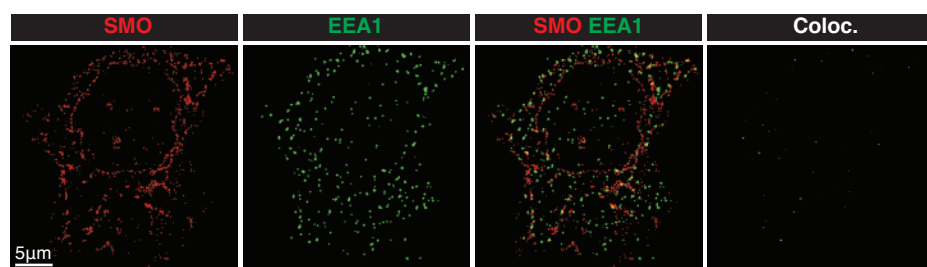

Supplement: S7 Fig — (A) Model showing the cellular compartments and their respective markers for analyzing SMO distribution. (B) Immunofluorescence showing costaining of C-term SMO–HA (red) with ERP72, RCAS1, SYNTAXIN-6, RAB7, and EEA1 (green). Scale bar = 10 μm. (C, D) N-term HA–SMO WT (upper panels) and A1 mutant (lower panels) costaining with ERP72 (C) and RCAS1 (D). Scale bar = 10 μm. (E) Western analysis showing HA–SMO WT and A1 expression levels in the ESCs clones. Actin is blotted as loading control. (F–H) SR microscopy distribution of SMO–HA (red) and ERP72 (F), RCAS1 (G) and RAB7 (H) (green) in the SR experiments shown in Fig 6A–6C. Scale bar = 5 μm. (I) Cellular localization of SMO–HA (red) and EEA1 (green) in SR experiments. Colocalizing events are shown in independent plots and labeled in gray; scale bar = 5 μm. ESC, embryonic stem cell; HA, hemagglutinin; SMO, Smoothened; SR, super-resolution; WT, wild-type. (PDF) [file pbio.3001596.s010.pdf]

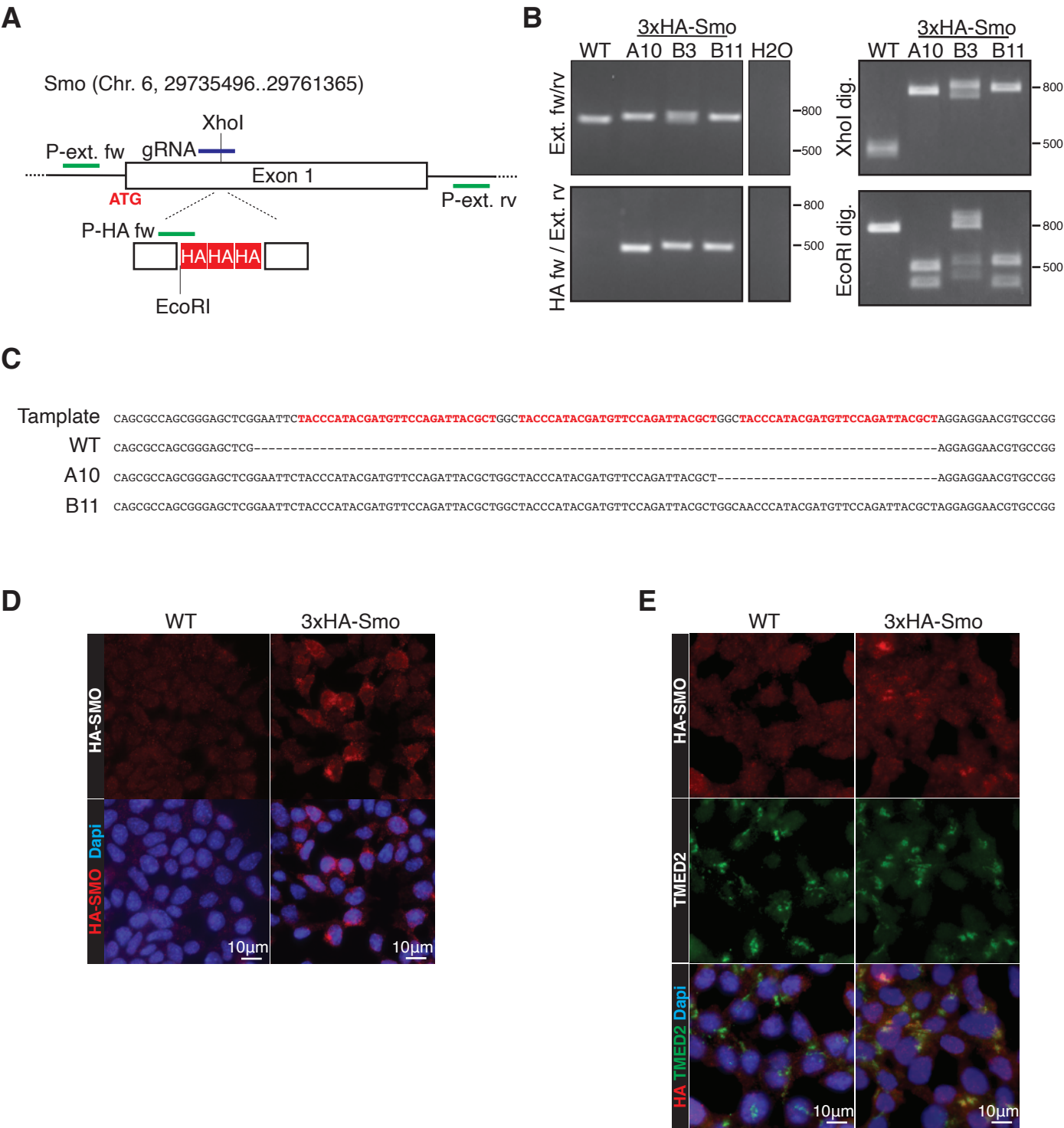

Supplement: S8 Fig — (A) Scheme showing the CRISPR/Cas-9 strategy used to introduce a 3xHA epitope tag into the endogenous Smo gene locus in ESCs. Location of primers and restriction sites used for screening positive clones are marked. (B) PCR analysis of WT and edited clones using primers flanking the edited region (top left) and specific for the edited region (bottom left). PCR products obtained using the external primers were digested with XhoI (present in WT cells, top right) and with EcoRI (introduced with the 3xHA epitope). All analyzed clones are characterized by the integration of the 3xHA sequence. The B3 clone shows a heterozygous genotype with just a single allele edited. (C) Sequence of the targeted locus is shown for WT cells and for A10 and B11 clones. (D) Immunofluorescence showing HA staining in WT and 3xHA–SMO ESCs. Scale bar = 10 μm. (E) Immunofluorescence showing colocalization of HA staining (red) with TMED2 (green) in WT and 3xHA–SMO ESCs. Cells were subjected after fixation to an antigen retrieval protocol specific for the detection of ER–Golgi resident proteins (see Materials and methods for details). Scale bar = 10 μm. ER, endoplasmic reticulum; ESC, embryonic stem cell; HA, hemagglutinin; SMO, Smoothened; WT, wild-type. (PDF) [file pbio.3001596.s011.pdf]

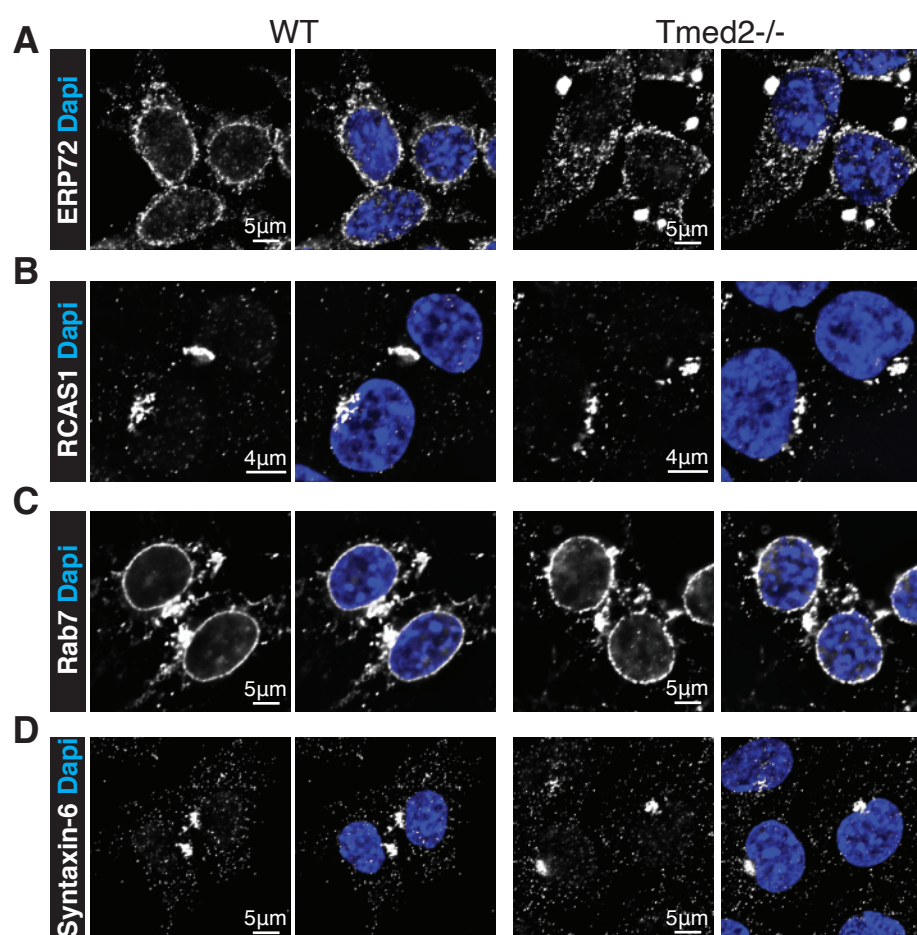

Supplement: S9 Fig — Immunofluorescence showing ERP72 (A), RCAS1 (B), RAB7 (C) and SYNTAXIN-7 (D) distribution in control (left panels) and Tmed2−/− (right panels) cells. Scale bar = 5 μm in (A), (C), and (D); scale bar = 4 μm in (B). (PDF) [file pbio.3001596.s012.pdf]

**A**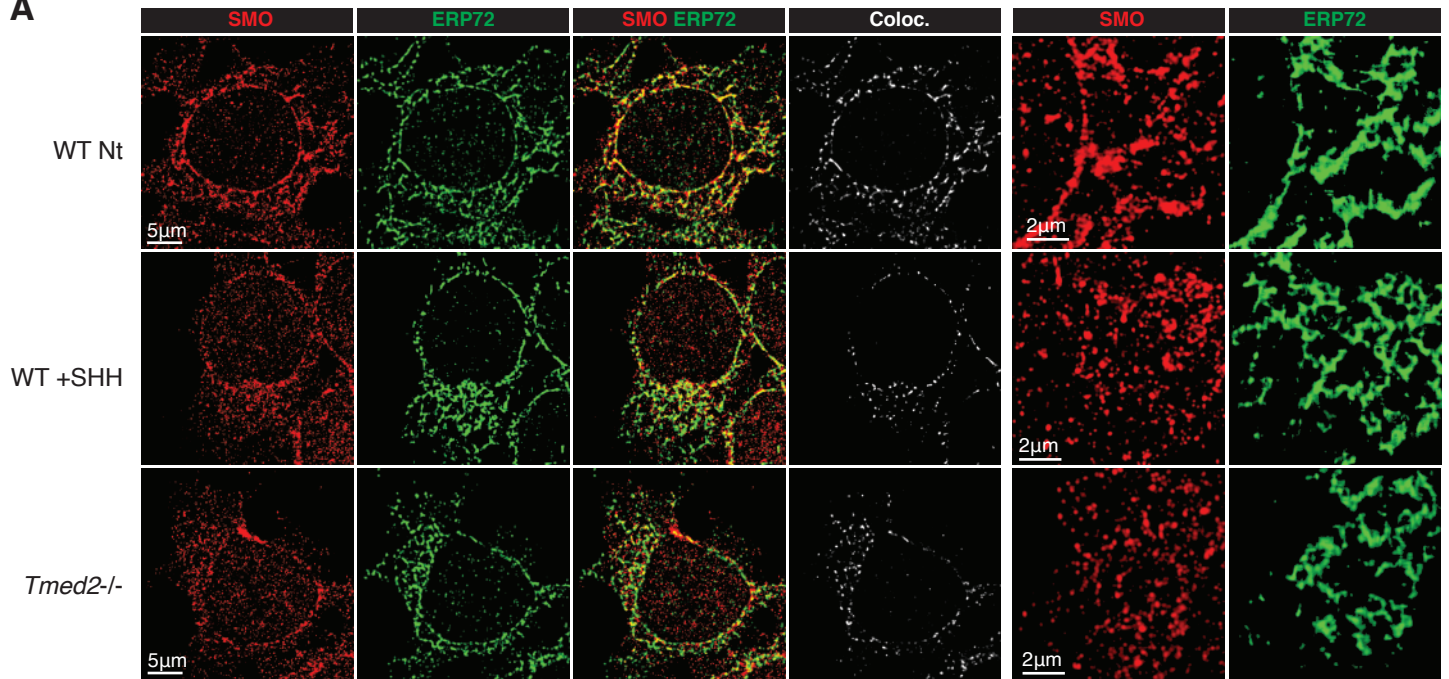**B**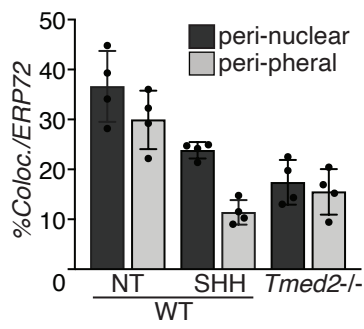**C**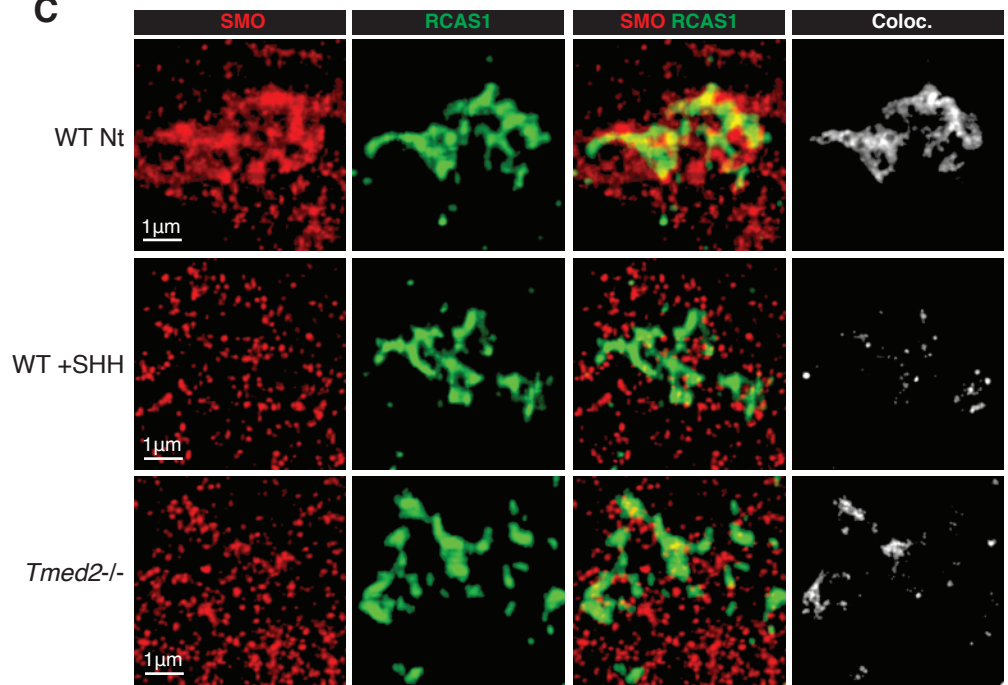**D**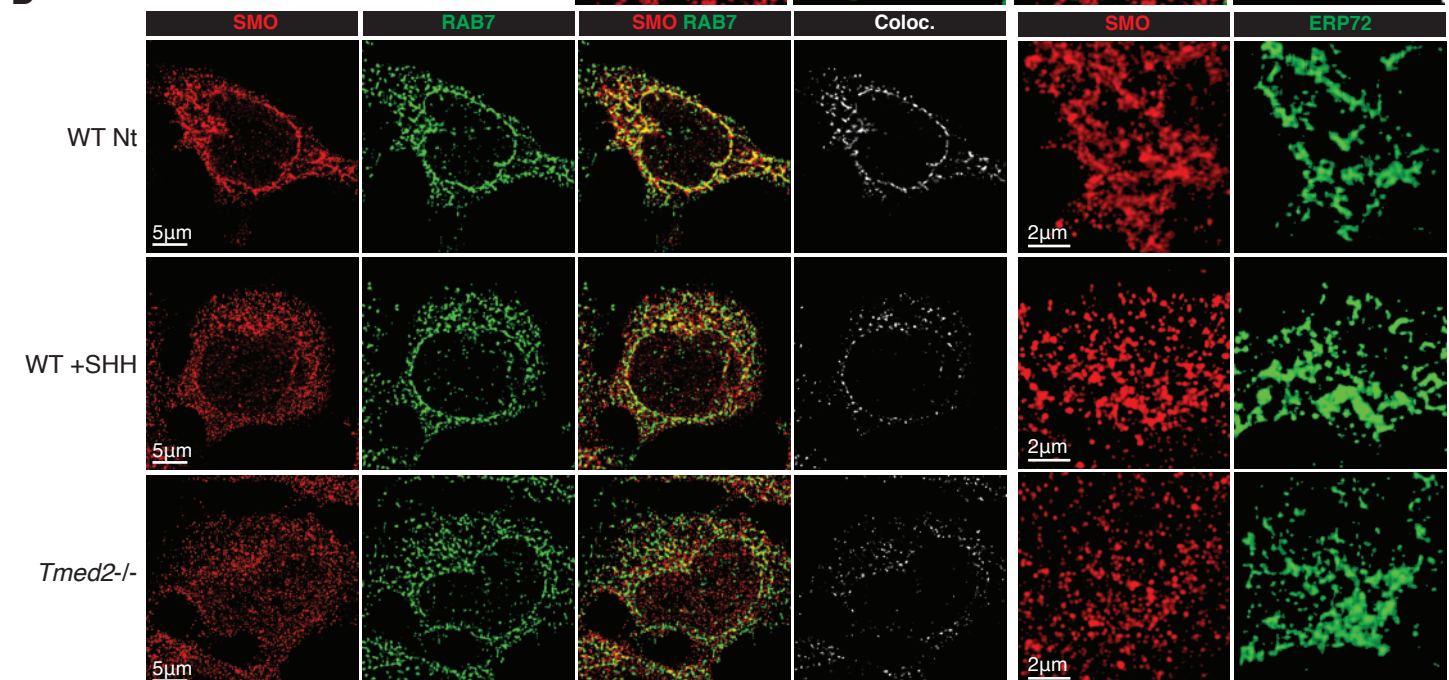

Supplement: S10 Fig — (A–C) SR SMO colocalization with (A) ERP72, (C) RCAS1 and (D) RAB7 in control (untreated and SHH treated) and Tmed2−/− NPCs. SMO–HA cellular distribution (red) was compared to organelle marker staining (green) in dual-color 3D-STORM experiments. Colocalizing events are shown in independent plots and labeled in gray; scale bar = 5 μm. Magnified area are shown on the left; scale bar = 2 μm. (B) Plot showing the percentage of SMO–HA colocalizing events relative to ERP72 localized in perinuclear or peripheral area upon SHH treatment and Tmed2 mutation. The data underlying all the graphs shown in the figure are included in the S1 Data file. NPC, neural progenitor cell; SHH, Sonic hedgehog; SMO, Smoothened; SR, super-resolution. (PDF) [file pbio.3001596.s013.pdf]

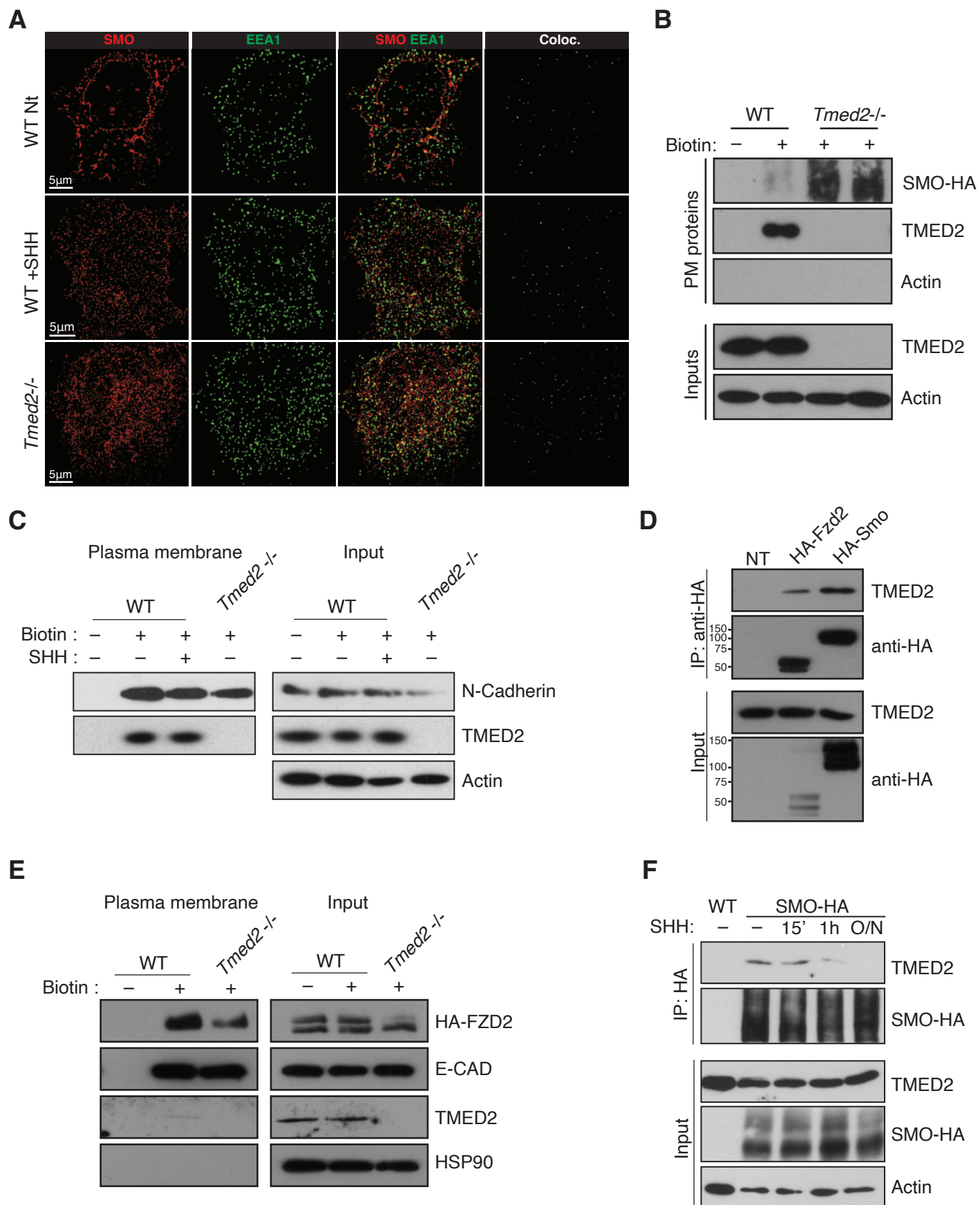

Supplement: S11 Fig — (A) SR SMO colocalization with EEA1 in control (untreated and SHH treated) and Tmed2−/− NPCs. SMO–HA cellular distribution (red) was compared to organelle marker staining (green) in dual-color 3D-STORM experiments. Colocalizing events are shown in independent plots and labeled in gray; scale bar = 5 μm. (B, C) Tmed2 regulates SMO localization at the PM. Cells were treated with NHS-SS-Biotin (Biotin) to label PM proteins, and with SHH as indicated. Western analysis of proteins present at the PM and input before purification (1/50 of pull-down) is shown. (D) The GPCR FZD2 binds to TMED2 in 293T cells. Western analysis of co-immunopurification of endogenous TMED2 with SMO–HA and FZD2-HA transfected in 293T cells (top) and input (1/25 of IP, bottom). (E) FZD2 secretion is not increased by the Tmed2 mutation. WT and Tmed2−/− ESCs overexpressing FZD2-HA were treated with NHS-SHHS-Biotin (Biotin) to label PM proteins. Western analysis of proteins present at the PM and input before purification (1/50 of pull-down) is shown. (F) Western analysis of co-immunopurification of endogenous TMED2 with SMO–HA (top) and input (1/25 of IP, bottom) in NSCs overexpressing C-term HA tagged SMO. Cells were treated with recombinant SHH for the indicated amount of time. Actin is shown as a loading control. HA, hemagglutinin; NPC, neural progenitor cell; NSC, neural stem cell; PM, plasma membrane; SHH, Sonic hedgehog; SMO, Smoothened; SR, super-resolution; WT, wild-type. (PDF) [file pbio.3001596.s014.pdf]
