# Supplementary material for: TMED2 binding restricts SMO to the ER and Golgi compartments
Source: PLoS Biol. 2022 Mar 30;20(3):e3001596. doi: 10.1371/journal.pbio.3001596 (PMC9000059; doi:10.1371/journal.pbio.3001596)
Supplement: S12 Fig — (PDF) [file pbio.3001596.s015.pdf]

Di Minin_S12 Figure

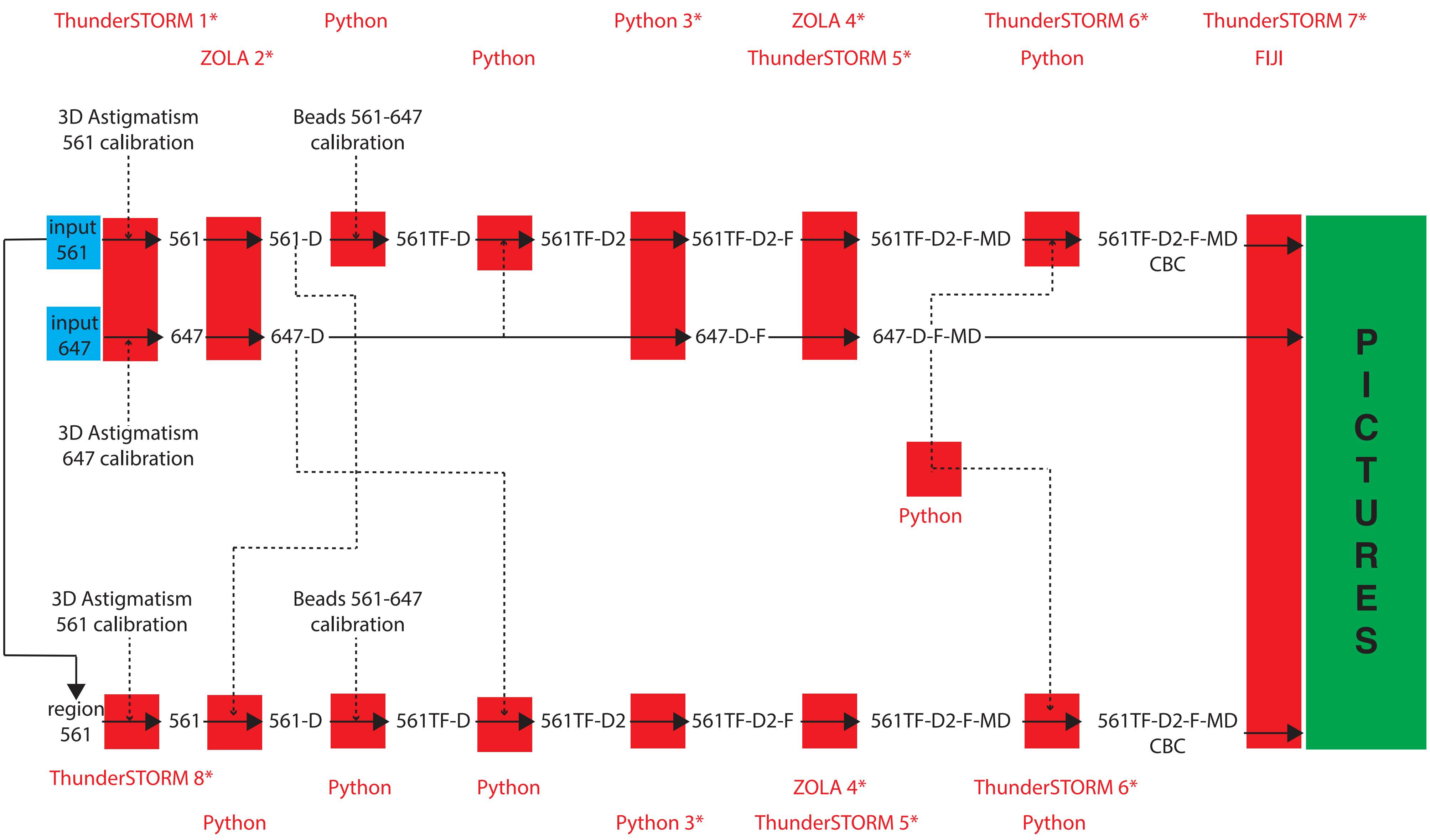

1* filter = [Wavelet filter (B-Spline)] scale 2.0 order=3 detector=[Local maximum] connectivity= 8-neighbourhood threshold=2*std(Wave.F1) estimator= [PSF: Elliptical Gaussian] sigma=1.6 fitradius=3 method= [WLs]
2* cross-correlation_pixel_size=50 number=5 maximun_drift=4
3* frame>2000 uncertainty_xy<50 uncertainty_z<200 -600<z [nm]<600nm
4* max._lateral=20 max._axial=40 off_frame=100
5* neighbors=10 radius=100.0 dimensio=3D
6* CBC addcbc=true radiustep=100 addnncount=false stepcount=3 dimension=3D addnnist=false
7* Gaussian 5 or 20 magnification. For 561 channel gamma=0.5
8* filter = [Wavelet filter (B-Spline)] scale 2.0 order=3 detector=[Local maximum] connectivity= 8-neighbourhood threshold=2*std(Wave.F1) estimator= [PSF: Elliptical Gaussian] sigma=1.6 fitradius=3 method= [WLs] MEFP=true nmax=5 pvalue=1.0E-6
