# Supplementary material for: TMED2 binding restricts SMO to the ER and Golgi compartments
Source: PLoS Biol. 2022 Mar 30;20(3):e3001596. doi: 10.1371/journal.pbio.3001596 (PMC9000059; doi:10.1371/journal.pbio.3001596)

Figure 1J

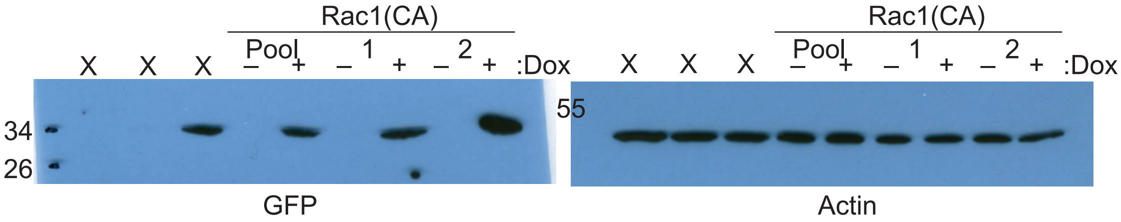

Figure S2K

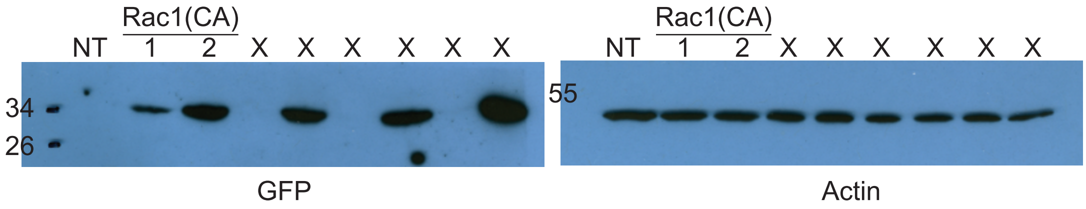

Figure S2L

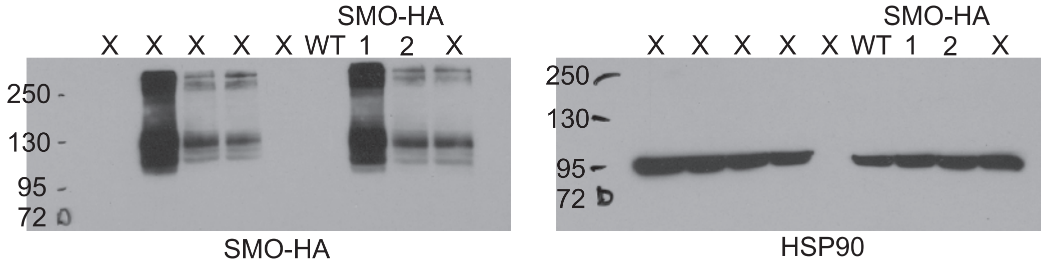

Figure 2C

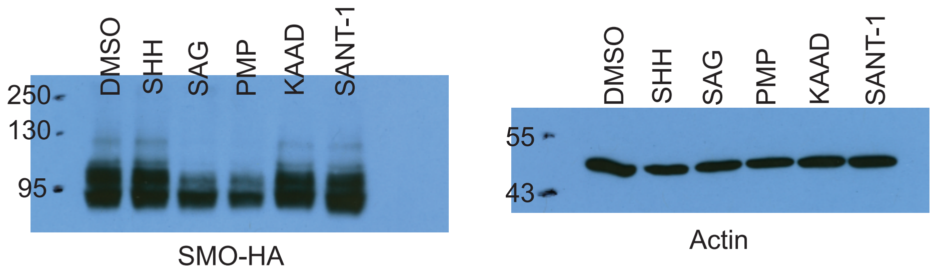

Figure 2D

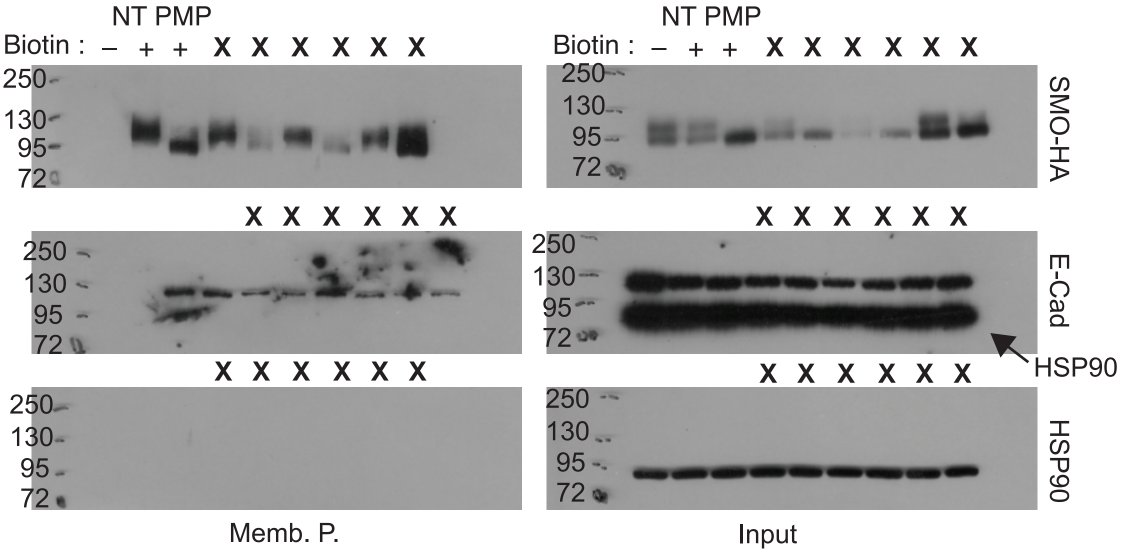

Figure 3B

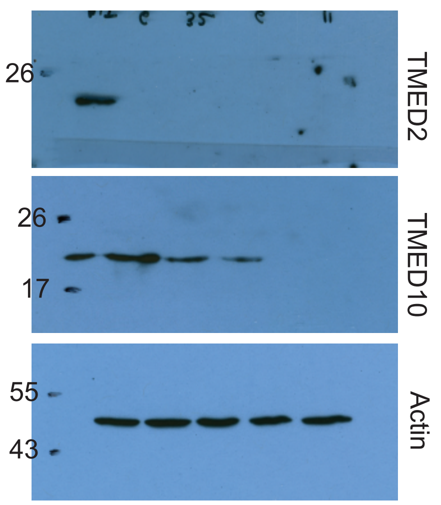

Figure 3G

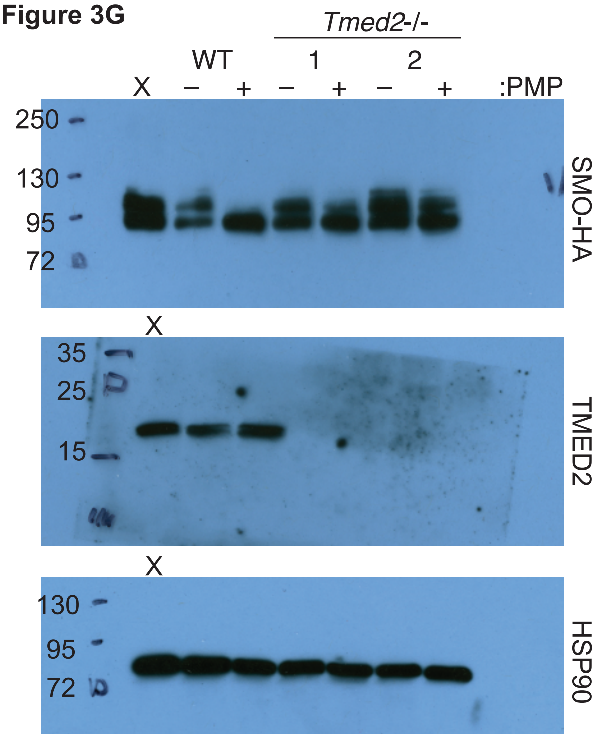

Figure 4A

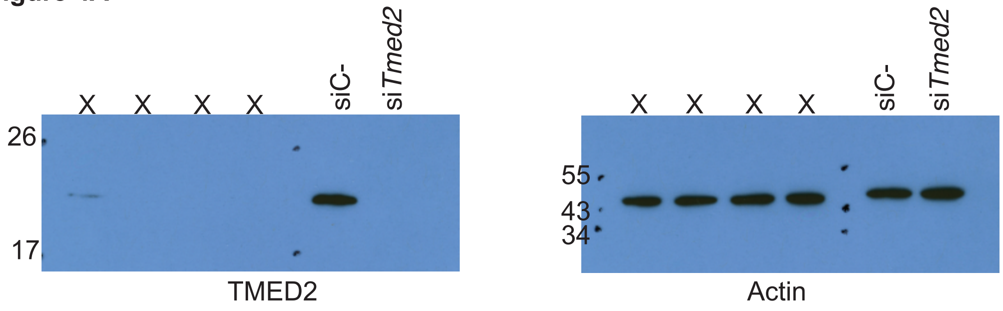

Figure 4G

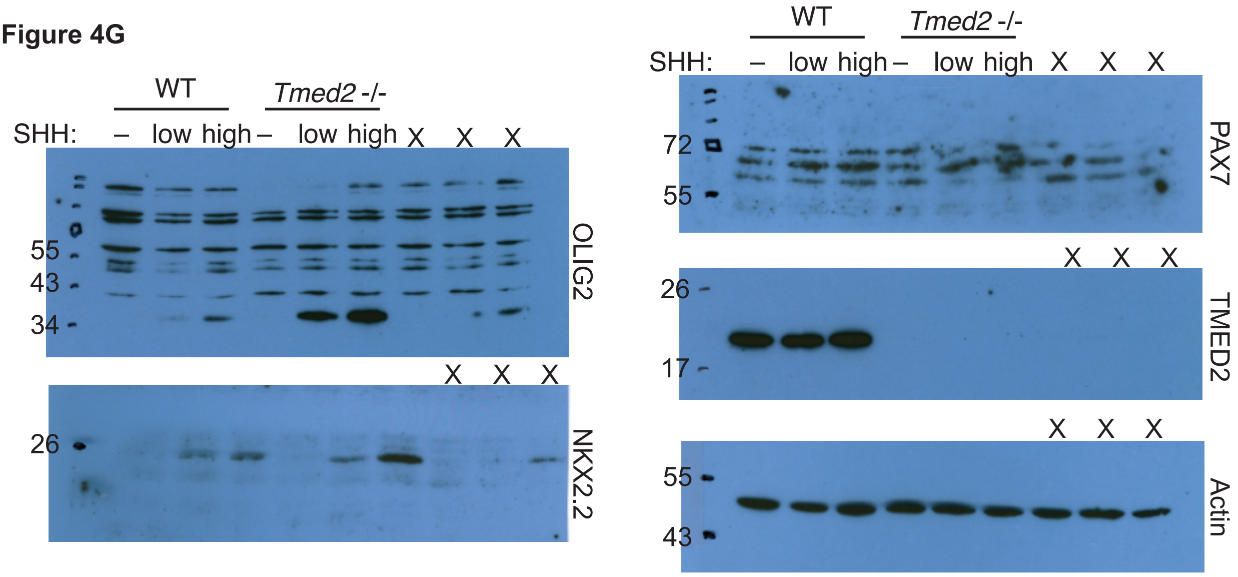

Figure S7E

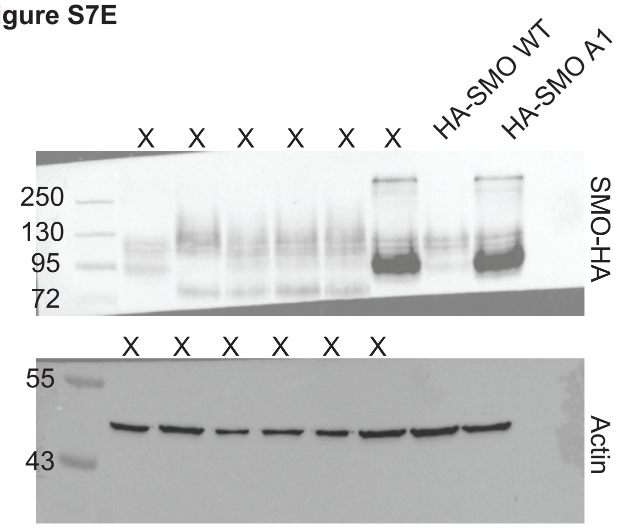

Figure S8B

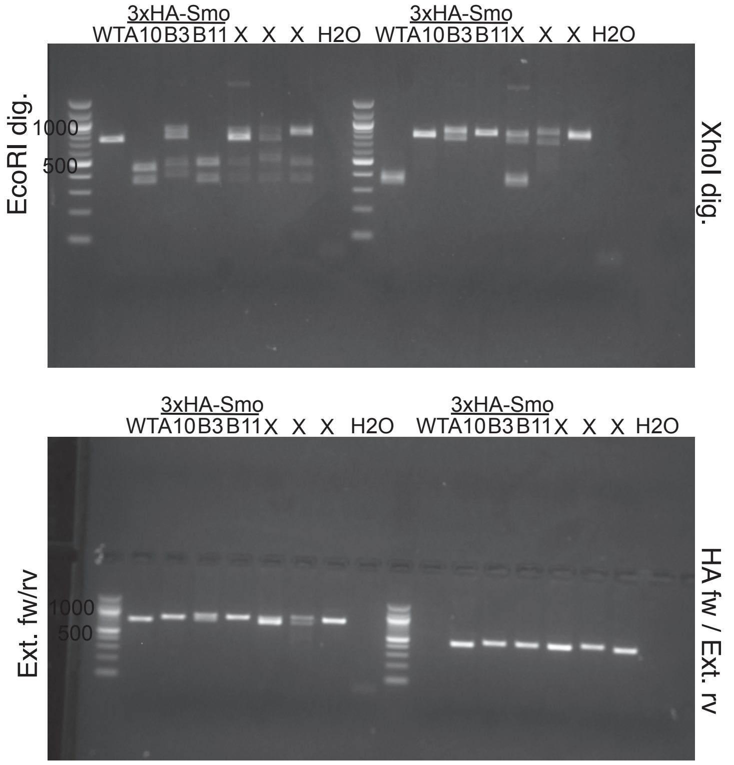

Figure 6F

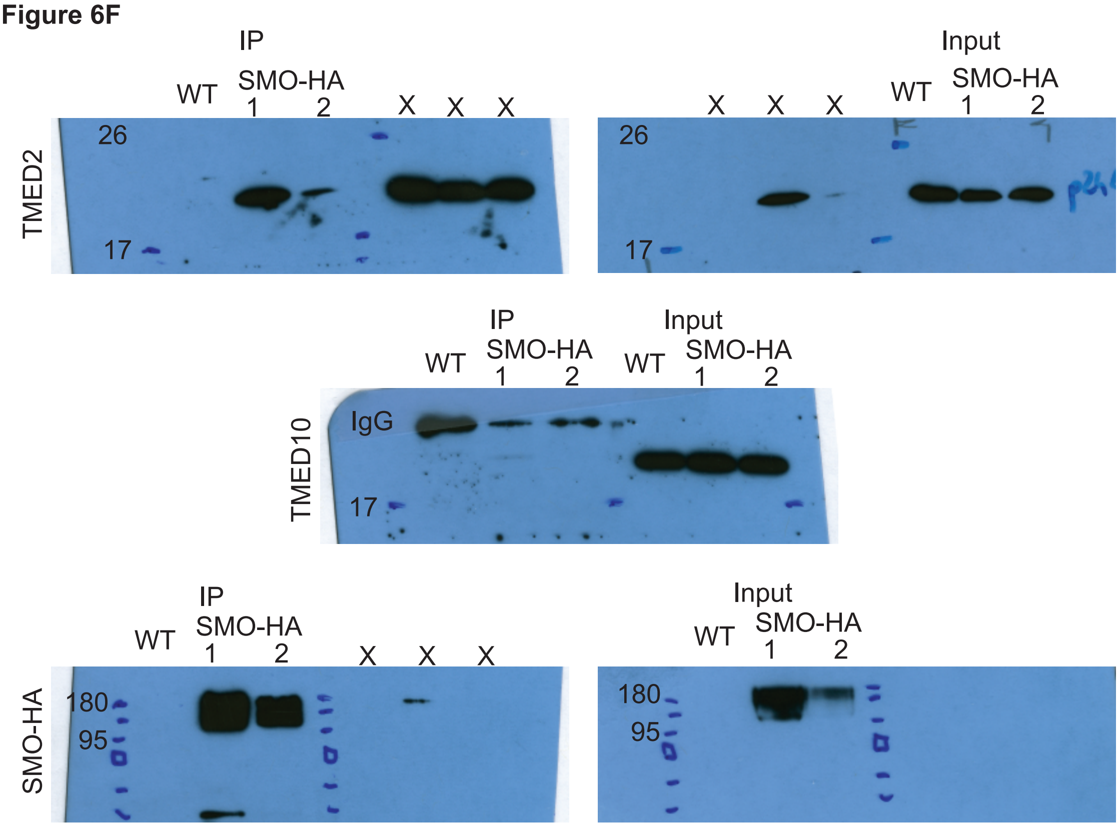

Figure 6G

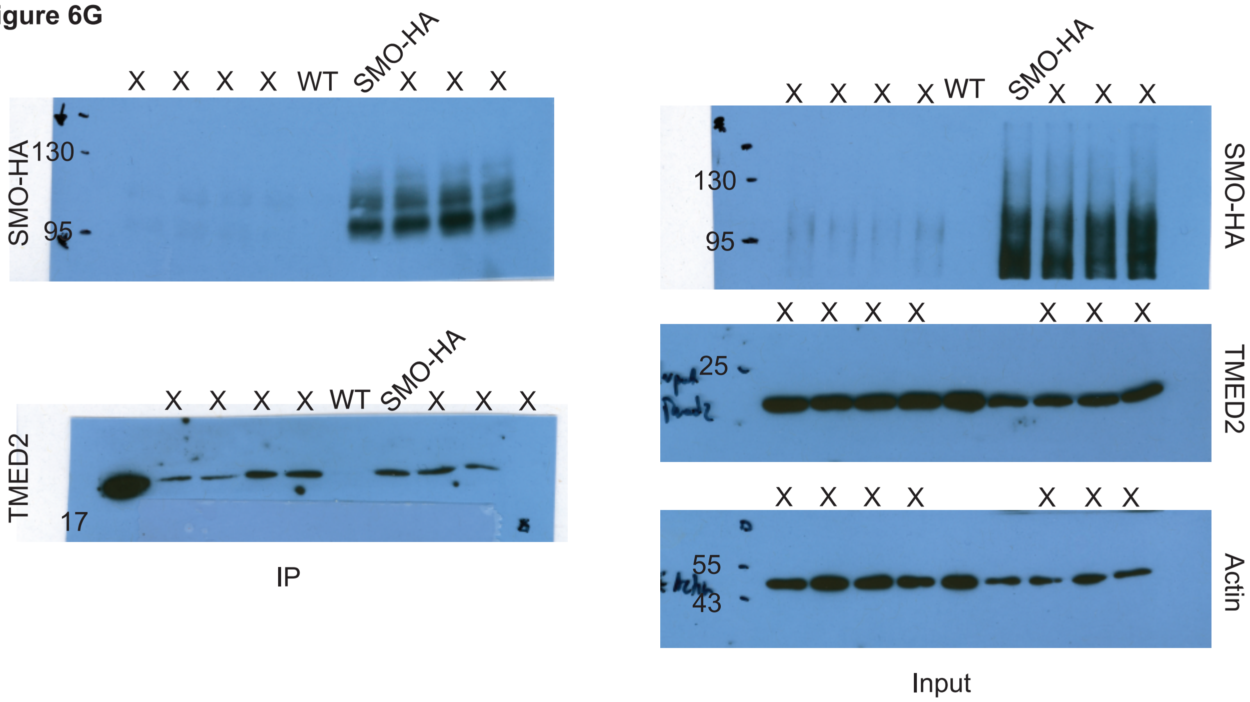

Figure 7G and S11C

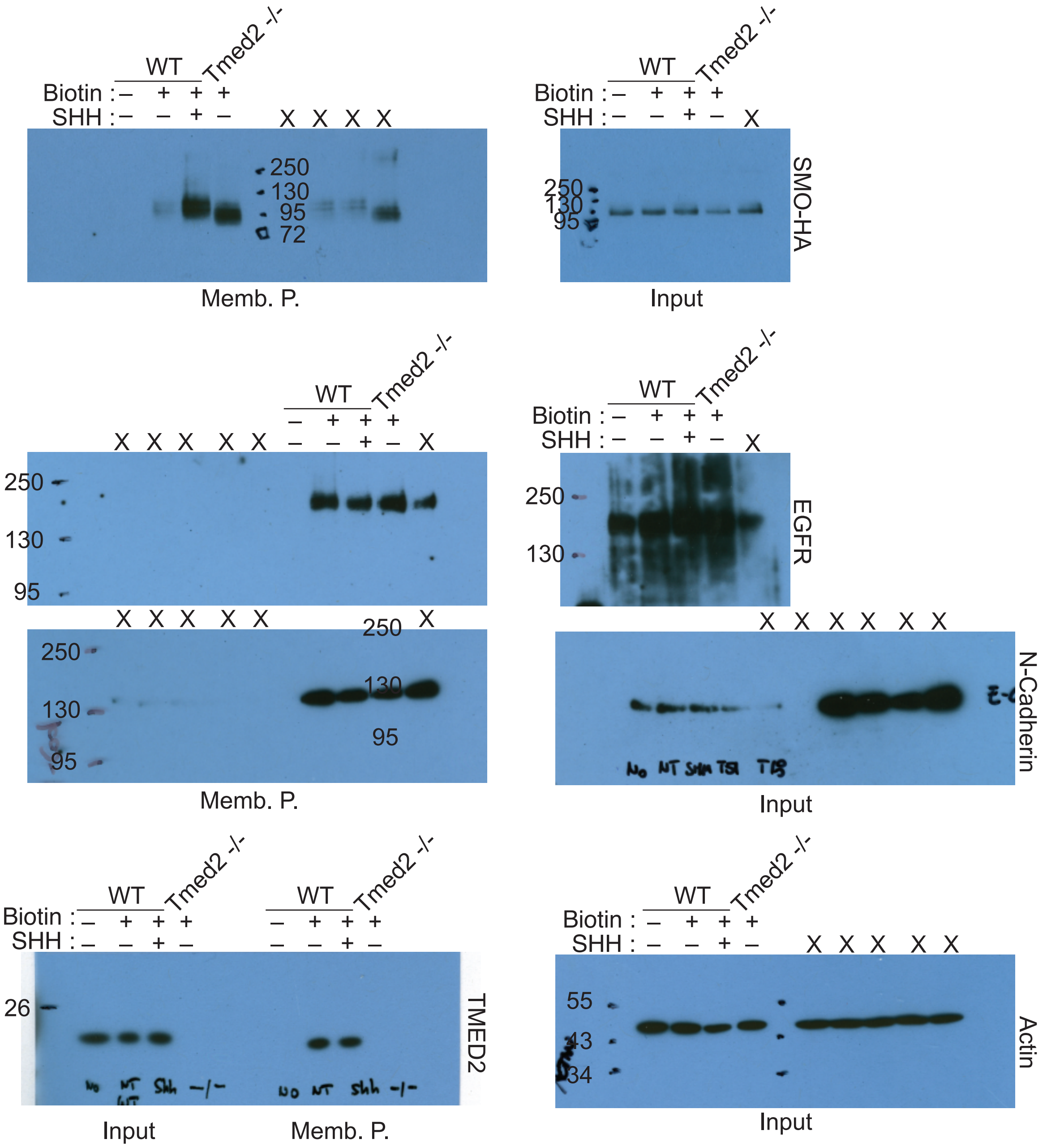

Figure S11B

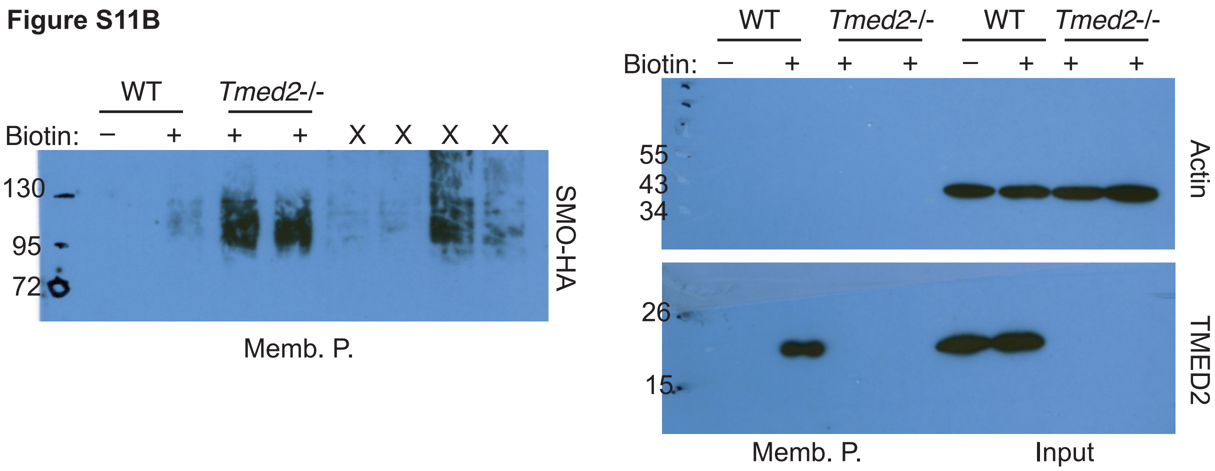

Figure S11D

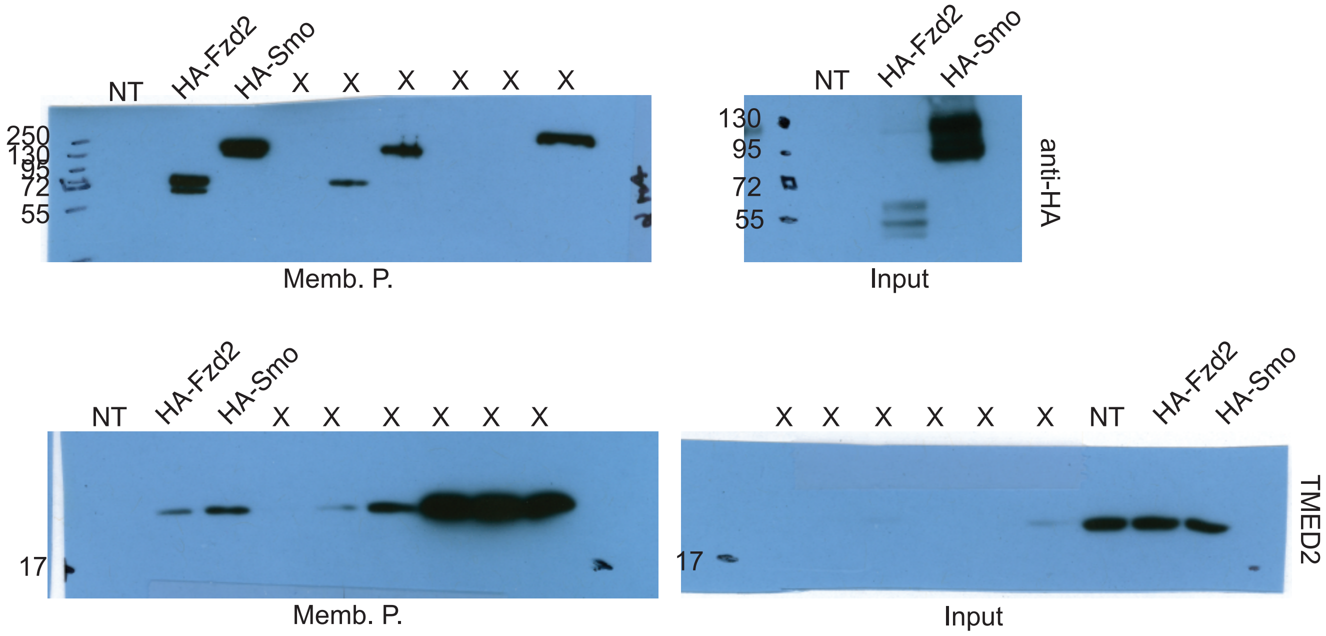

Figure S11E

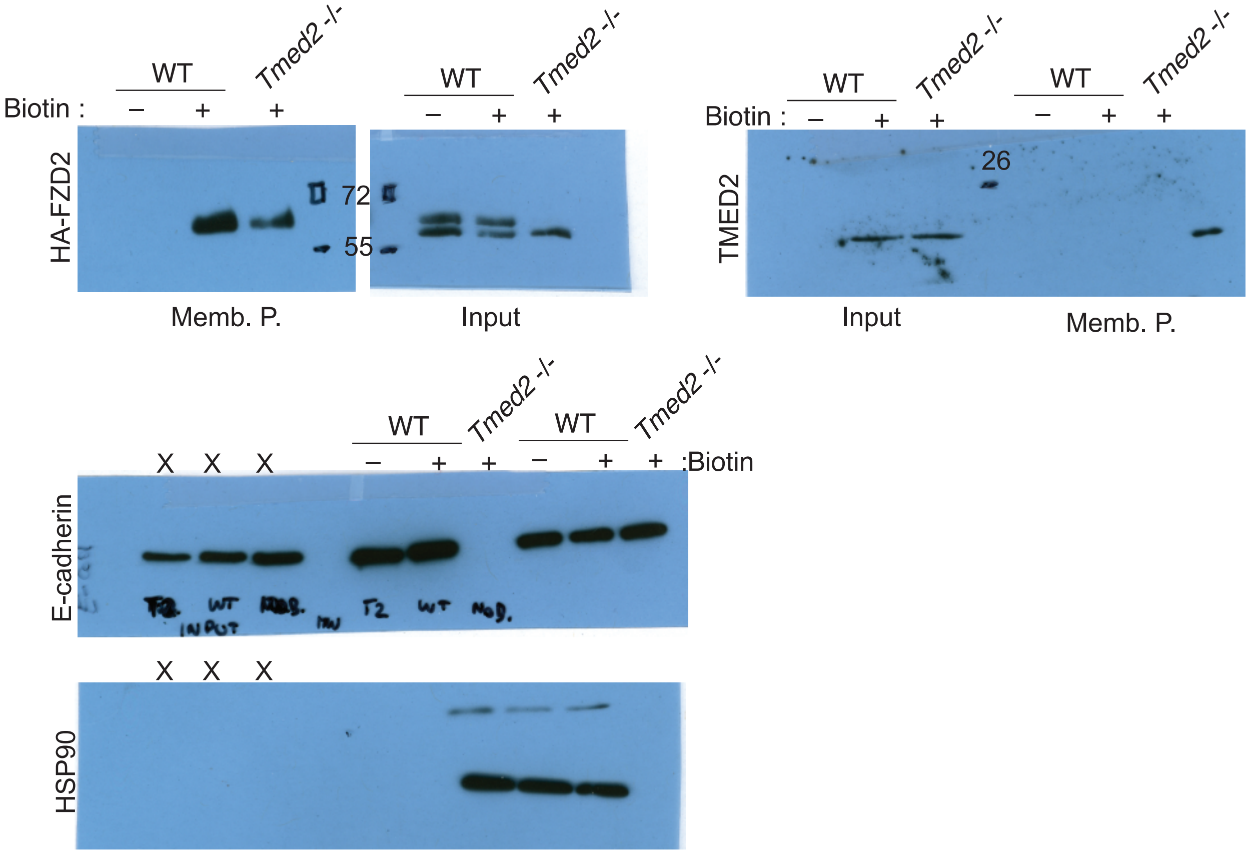

Figure 7I

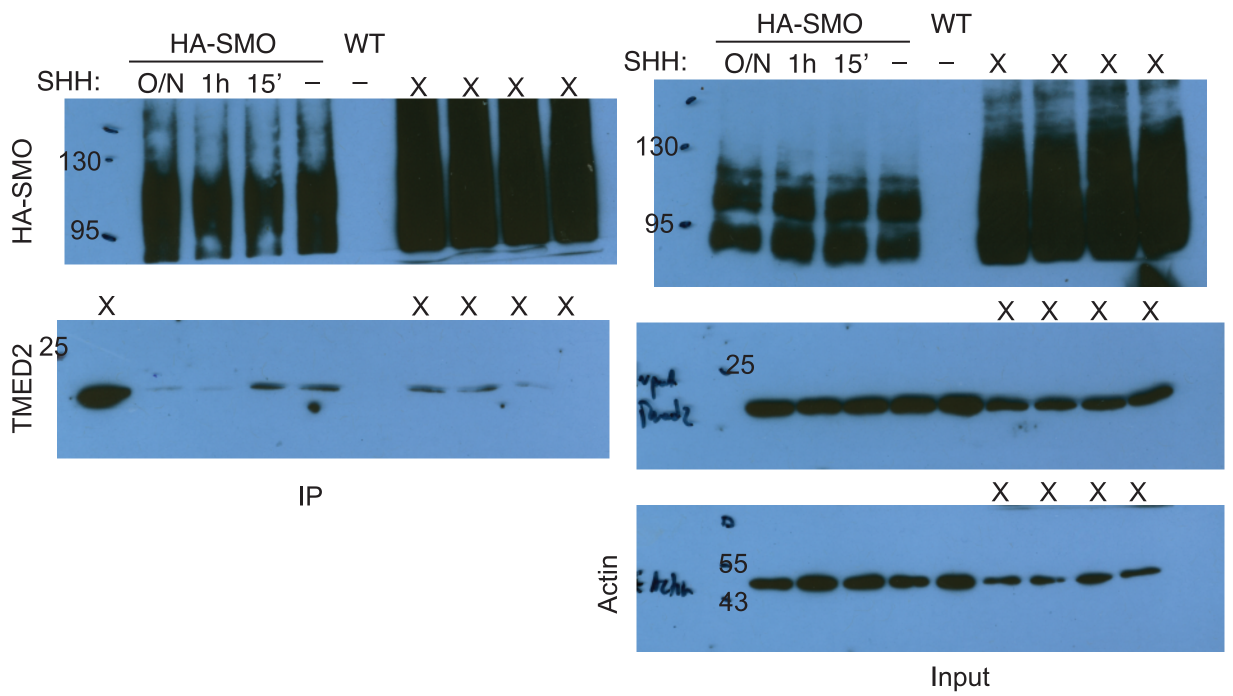

Figure S11

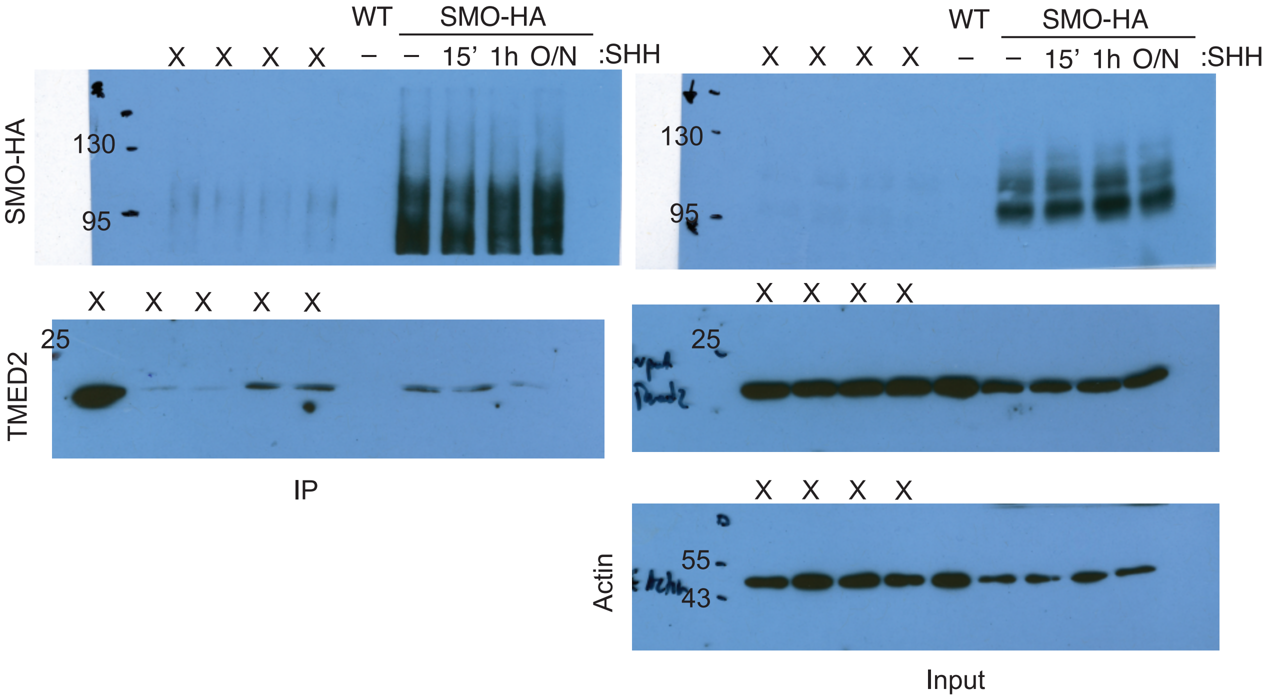

Supplement: S1 Raw Images — (PDF) [file pbio.3001596.s016.pdf]
