# Supplementary material for: TMED2 binding restricts SMO to the ER and Golgi compartments
Source: PLoS Biol. 2022 Mar 30;20(3):e3001596. doi: 10.1371/journal.pbio.3001596 (PMC9000059; doi:10.1371/journal.pbio.3001596)
Supplement: Attachment — Submitted filename: point_to_point_response.pdf [file pbio.3001596.s018.pdf]

Response to the editorial comments

Response: We would like to thank the reviewers and the editor for their comments, which have helped us to further strengthen our conclusions. Before we respond to the individual points raised by the reviewers, we would like to address the specific issues pointed out in the decision letter.

1. The editor points out the issue if Tmed2 function is specific for Smoothened or a wider spectrum of GPCRs could be similarly regulated. This concern was raised by reviewer 1 and 3. We would like to respond in the following way. Tmed2 has been associated with GPCRs before and a function and trafficking has been proposed. We have cited these studies in our original manuscript. Smoothened trafficking has not been studied before and Tmed2 has not been implicated in signalling before our screen. In the course of the revision we have investigated if a related GPCR Fzd2 would also interact with Tmed2 and if it was similarly restricted as Smoothened from the plasma membrane. Our new data show that we can detect a biochemical interaction using epitope tagged Fzd2 (new Suppl. Fig. S11D). In contrast to Smoothened, Tmed2 does not restrict Fzd2 from reaching the plasma membrane (new Suppl. Fig. S11E). These data show that likely Tmed2 functions in glycosylation and quality control of Fzd2. Therefore, we conclude the interaction between Tmed2 and Smoothened stands out as it affects trafficking to the plasma membrane and is regulated by Hedgehog signalling. We believe the identification of Tmed2 as a transport factor for Smoothened and defining the mechanism in modulating Smoothened function identifies a specific step in mammalian Hedgehog signalling. The observation the also other signalling receptors interact with Tmed2 provides an additional opportunity to investigate its wider function for instance in planar cell polarity in the future.

2. A major concern raised by reviewer 1 has been the question if our expression of Smo-HA at 5 to 10 times the endogenous level might have introduced artefacts and specifically if the apparent restriction of Smo to the ER-Golgi might be a non physiological consequence of overexpression. We did investigate a Smo mutant that has a single amino acid substitution and is constitutively active in our original manuscript. Constitutively active SmoA1 is not restricted by Tmed2 and did not accumulate in the Golgi compartment even if overexpressed to high levels. Our earlier attempts to detect Smo using antibodies in cell internal compartments were unsuccessful, which is likely related to the low abundance of the protein or the signal to noise ratio of antisera. For addressing the question further we include in the revised version an experiment in which we have targeted a 3xHA tag to the endogenous Smo gene (new Suppl. Fig. S8 and new Fig. 6H). After selection of a highly specific anti-HA antiserum, we could detect Smo at the endogenous level in IF experiments (new Suppl. Fig. S8 and new Fig. 6I). From these new data we can make the following observations (page 15, second paragraph). Firstly, the distribution of Smo protein at the endogenous level closely resembles the one that we have observed earlier with our expression construct. Secondly, endogenous Smo-HA colocalizes with Tmed2 (new Fig. 6I). Thirdly, we notice that about 30% of cells express endogenous Smo at a level that is sufficiently high to be detected. This is in contrast to our Smo-HA expression construct where we observe the large majority of cells express Smo-HA. This last observation has led us to reassess our earlier findings about a synergistic effect of the Tmed2 mutation with Smo-HA overexpression leading to an apparent activation of HH signalling in our neural differentiation experiments. We believe that increased transcription of the ventral marker Olig2 in the Tmed2 mutant Smo-HA expressing cells is due to the higher number of SMO expressing cells that can be targets of low SHH produced within the culture system (original Fig. 7H). The Tmed2 mutation will

increase the response leading to an observable increase in the number of ventral marker expressing cells. This is also consistent with our new data that ventral markers are not detectable on the protein level in the absence of exogenous SHH (new Fig. 7H). Therefore, SMO-HA from our expression construct recapitulates the endogenous SMO to a large extent with only small differences. Importantly, transgenic SMO-HA is under the same restriction by Tmed2 as the endogenous SMO protein. We hope that our new data will be convincing that Tmed2 is a regulator of endogenous Smoothened and physiologic Hedgehog signalling.

3. We thank reviewer 3 for the helpful comments and for suggesting Dbx1 as an additional marker for analysing the ventralization phenotype that we observe in neural tubes of Tmed2 mutant mice. We have performed additional experiments which have allowed us to further strengthen our conclusion of a ventralizing effect of the Tmed2 mutation in the neural tube. We now show the expression of Dbx1 in neural tube sections (new Fig. 5D and new Suppl. Fig. S6D). The Dbx1 domain appears expanded and in direct contact with the NKX6.1 domain in the Tmed2 mutant, whereas a clearly defined and separate stripe of Dbx1 is seen in the wild type. The more dorsal extension of Dbx1 if further consistent with a ventralizing effect of the Tmed2 mutation. In reassessing the mutant phenotype we realized that the markers in Tmed2 mutant neural tubes closely resemble the Gli3 mutant, and not Ptch1 or Sufu mutant neural tubes. This observation is consistent with an increase in Hedgehog signalling strength but not ectopic activation of the pathway in Tmed2 mutant neural tubes. We discuss this in the revised text on page 14, first paragraph an discussion on page 20.

Response to the comments raised by reviewer 1

Rev. 1:
This manuscript 1) outlines a screen performed to identify new modulators of Sonic Hedgehog (SHH) signaling, and 2) examines functionality of a hit from the screen (TMED2) in regulation of SHH signal output in vertebrates. The authors provide cell biological and genetic data suggesting that TMED2 plays a negative regulatory role for control of SHH by preventing exit of the signal transducing component Smoothened (SMO) from the Golgi. Identification of TMED2 and a related protein TMED10 as modulators of SHH signaling will be of interest to some in the field. However, concerns about experimental design and data interpretation limit enthusiasm for the work and make it unsuitable for publication in its current form. Several experiments lack appropriate controls, validation of the screen is insufficient, and conclusions are over-stated. Primary concerns are that the screen is not well validated, several experiments lack controls, and results are over-interpreted. It is also a confusion for this reviewer that the authors screen for 'noncanonical' SHH signaling activities, then investigate a hit from this screen by examining canonical signaling.

Response: We thank the reviewer for the comments which have allowed us to improve our manuscript. We note that a confusion has arisen over our description of HH pathway components that act upstream of ciliary processing of Gli factors. Our intention was to contrast our newly discovered factors from recent screens which have advanced on the pathway downstream of Smoothened. We would like to point out that our mutations deregulate the HH pathway upstream of (and through) Smoothened. Therefore, we expect our mutations to affect canonical and non-canonical outcomes, which we show in our study. However, we also need to point out that the canonical pathway is not functional in embryonic stem cells and, hence, in this system we observe only effects of non-canonical signalling. We regret that our original description was confusing and we have provided clarification in the revised text (page 19, end of first paragraph, and discussion page 20).

Specific points to be addressed are listed below.

1. The authors observe that embryonic stem cells (ESCs) that lack SMO have a slower growth rate and undergo apoptosis at a higher rate than control cells. They hypothesize that SMO plays a role in preventing death of pluripotent cells. The authors investigate whether this is a canonical (GLI dependent) or noncanonical (GLI independent) signal by looking at qPCR of SHH gene targets Gli1 and Ptch (Figure 1). Given that Gli1 and Ptch are unaffected by Smo loss or small molecular modulators in ESCs, the authors conclude that defective canonical signaling is not leading to cell death. However, Gli1 and Ptch may not be reliable indicators of all the genes induced by SHH signaling. Phenotypes could be result from altered regulation of other target genes. What happens if you do similar treatments in the presence of a Gli antagonist (GANT)?
Response: We have performed new experiments and show treatment of ESCs with GANT61 (Suppl. Fig. S1E and 3T3 control in S1F). We observe no effect of 1 micromolar GANT61 on ESC viability and growth, the same concentration is able to block SHH induction of Gli transcription in 3T3 cells. These results support our previous conclusion that loss of canonical HH signalling targets are not the cause of cell death in ESCs.

2. Figure 1E-F, S1C - how many replicates are represented by the data? Do the dots equal three biological replicates or technical replicates? Please provide this information in the figure legends.

Response: We have clarified that the experiment was performed with 3 biological replicates in the figure legends of Fig. 1E and Suppl. Fig. S1C-D.

3. Figure 1G - The authors should indicate whether cyclopamine treatment decreases survival.
Response: The effect of KAAD-cyclopamine was shown in Fig. 2B of the original submission. We have used KAAD-cyclopamine instead of cyclopamine for technical reasons. It is reasonable to assume that KAAD-cyclopamine has similar effects as cyclopamine. We clarified in the figure legend that KAAD stands for KAAD-cyclopamine.

This experiment is needed to support that PTX effects are due to compromised G alpha i (Gi) signaling downstream of SMO. What happens if you pre-treat with SHH? Does PTX have an effect in Smo -/- cells?
Response: We have performed a new experiment showing that Smo-/- ESCs are not further affected by PTX treatment (new Fig. 1H). This is consistent with our conclusion that PTX acts via repression of Smo mediated Gi activity.

4. Figure 2C - The authors are using SMO staining in the 'periphery' to estimate membrane localization. Performing extracellular staining on non-permeabilized cells would be a clearer way to visualize plasma membrane SMO.
Response: We have performed an analysis of membrane proteins after PMP treatment (new Fig. 2D). Our new data show that the abundance of SMO at the plasma membrane is decreased after treatment with high concentration of Purmorphamine. In addition, under these condition SMO appears at a lower molecular weight suggesting a lack of post-translational modifications. Notably, the loss of the higher molecular weight band of Smo is already observed in the input, which reflects the total Smo protein. We believe that this likely reflect a loss of glycosylation by a change in the route Smo takes in the presence of PMP. The reviewer's comments have alerted us to this important aspect of Smo regulation. We have included route selection as a consideration in the discussion but cannot at the moment provide a comprehensive analysis. We would like to leave this to future studies.

5. Figure 2D -The authors state that they see less SMO on the membrane after treatment with high SAG or PMP. They make the assumption that cell surface SMO is reduced because they do not see as much higher-molecular weight glycosylated SMO following drug treatment. Glycosylation is not a clear indicator of plasma membrane localization. The results provided do not properly demonstrate this. Cell surface biotinylation would be more informative biochemical technique to use.
Response: We have performed the experiment as requested by the reviewer (see response to point 4 above) and fully agree that the glycosylation status does not clearly reflect membrane localization. Indeed our new data show that the "non-glycosylated" form is present on the plasma membrane in PMP treated cells (new Fig. 2D).

6. The authors use super-physiological concentrations of SAG and PMP in the screen and validations, so there is concern they are validating off-target effects.
Response: We agree with the reviewer that it is important to show that SAG and PMP are actually affecting the Hh signaling pathway and act through SMO in our experiments. To address this concern we have performed a number of control experiments in our study. We show that the effect of PMP on survival of ESCs is largely removed in Smo-/- ESCs (Suppl. Fig. S2F). Overexpression of SMO-HA rescues cell death of ESCs after PMP treatment (Fig. 2E). In addition, treatment with ROCKi (Suppl. Fig. S2H), or expression of constitutively

active Rac1(CA) prevent PMP induced cell death (Suppl. Fig. S2J-K). In the course of our revision we have also investigated if PMP treatment induces myosine hyper-contractility as we have inferred from the observation of membrane blebbing and the rescue with ROCKi. We observe intense myosin light chain 2 phosphorylation at the cell cortex using a specific antiserum in ESCs after treatment with PMP. This effect is blocked by the Tmed2 mutation (new Suppl. Fig. S2I). Our data show that PMP causes a specific defect in contractility regulation in ESCs and this is counteracted by SMO. Together with the literature showing SMO is a target of PMP and SAG, these findings strongly suggest that the Hh pathway is affected in our experiments.

7. Figure 3E - differences in SMO localization minus and plus Tmed2 are unconvincing.
Response: We have performed immunoblot analysis of SMO after PMP treatment and show the data in new Fig. 3G. We find that the high molecular weight band of SMO is lost in wild type cells after PMP treatment, whereas it is maintained at a reduced level in Tmed2 mutant cells.

8. Figure 4A - TMED2 depletion is only having an effect in the presence of SHH? What happens following Tmed2 knockdown in the absence of SHH? Does basal activity come up? If not, is it a true negative modulator?
Response: From our results, we believe that Tmed2 regulates trafficking of Smo to the plasma membrane. Our data show that in Tmed2 mutant cells there is no elevation of basal Gli activity. Only in the presence of Hh ligand a stronger Gli response compared to wild type cells is observed (Fig. 4A, 4E, 4F and 4G). The higher amount of SMO at the membrane results in Tmed2 mutant cells is likely the reason for the elevated response. Our data show that the Tmed2 mutation does not lead to ectopic activation of Gli factors. Therefore, a second regulatory step for Smo entering the cilia is unaffected by the Tmed2 mutation. In the absence of HH ligands, loss of Tmed2 leads to an increased abundance of SMO at the plasma membrane but not at the cilia.

9. Page 12, line 11 - the statement that TMED2 is regulating canonical signaling based upon the results that SMO movement into primary cilia is unaffected by Tmed2 knockdown doesn't make sense. SMO entry into the primary cilium controls canonical signaling. Perhaps signaling to GLI is impacted, but the authors should not use the phrase 'canonical signaling' based on this result. Also, why is something that you pulled out as being part of the noncanonical response (based upon your screening strategy in ES cells) affecting GLI?
Response: We thank the reviewer for the comment and agree that we need to clarify our statement. We explain this now considering that the Tmed2 mutation acts upstream of and regulates SMO (discussion, page 20). The effect is a higher abundance of SMO at the plasma membrane already before a Hh signal is received. This has an immediate effect on (non-canonical) GPCR mediated Smo functions but would not affect ciliary Gli processing before a HH signal is received. We show in Fig. 4B that SMO is not localized at the primary cilia after depletion of Tmed2. SMO requires an additional signal - likely through Patched - to enter the ciliary compartment and affect processing of Gli factors. This is observed when Tmed2 mutant or depleted cells are treated with SHH through an elevated Gli transcriptional response (Fig 4A; Fig. 4B also shows that SMO localized at the primary cilia in Tmed2 depleted cells after SHH treatment). We have revised text on page 12, end of 1st paragraph, and page 14, first paragraph, and more clearly state our interpretation that Tmed2 acts upstream of and hence affects both canonical and non-canonical Hh functions, but there is no ectopic activation of Gli factors but a stronger HH signal dependent response in the Tmed2 mutant.

10. Figure 4C - this panel is not very informative because there is no control with TMED2 mutant neural tubes to support staining is specific.
Response: We apologize for not including a staining control for the histological sections in our original submission. We now show the staining of Tmed2 mutant neural tube sections in new Suppl. Fig.S5E (cited in text page 12 line 12). This experiment has further confirmed the specificity of the Tmed2 antiserum in addition to our IF and WB data from ESCs and neural progenitors (WB: Fig. Fig. 3B, 3G and 4G; IF: Fig. 6D)

11. Figure 4E - need to show the TMED2 staining to confirm the EBs are really knockout tissue.
Response: We show a WB of the Tmed2 mutant cells from which we derived the neuralized EBs in Fig. 3B, 3G, and 4G, which confirms the absence of Tmed2 protein.

Also, please show TMED2 knockouts without SHH stimulation. Again - it's unclear if changes are only occurring in the presence of SHH or if basal pathway signaling is enhanced in the absence of TMED2
Response: A quantification of marker expression in the absence of Hh was included in our original submission in Fig. 4F (black circles, Nt; non treated). Since there is no difference in the absence of SHH to wild type cells, we did not include the staining of the Tmed2 mutant in the figure. As explained above we detect elevated marker expression after SHH treatment but not before treatment in Tmed2 mutant neural EBs. This indicates a second regulated step for SMO to enter the ciliary compartment. Therefore, our conclusion is that the Tmed2 mutation alone does not cause ectopic activation of the Gli factors. We do expect elevated and/or ectopic GPCR activity of SMO in Tmed2 mutants based on our data in ESCs.

From panel G, it looks like signaling is not increased in the absence of SHH. What might this mean?
Response: We propose Tmed2 as a regulator that acts upstream of SMO, which points to an early regulated step in the Hh signaling cascade. However, we would like to point out that this is not the only regulated step. From our data it is clear that ciliary entry of SMO is regulated downstream of Tmed2, and ciliary entry is dependent on a SHH signal, thus, Patched dependent. Although, the Tmed2 mutation does not activate Gli activity by itself, it leads to a higher amount of SMO at the plasma membrane, which causes an elevated Gli response after stimulation with SHH. We have rephrased the text to now indicate that Tmed2 regulates the non-canonical GPCR function of Smo and modulates the canonical outcome of Gli activity after SHH treatment (page 12 end of 1st paragraph, and page 19, paragraph starting line 4, and discussion, page 20).

12. The labeling on panel 4E is unclear. What is the genotype of the EB that is one up from the bottom?
Response: We thank the reviewer for pointing the incomplete labelling of the figure out. We have modified the figure to clearly indicate the genotypes.

13. Figure 5 - Embryos of different developmental stages were compared between Tmed-/- animals and WT embryos to account for developmental delays/size differences. It is not appropriate to make this comparison because lots of patterning changes occur between E10.5 (mutant) and E9.0-9.5 (WT examples). This leads to concern that conclusions based on differences between WT and mutant neural tubes are not valid.

Response: We agree with the reviewer that it is difficult to compare the Tmed2 and wild type embryos. We have included the stage matched sections in Suppl. Fig. S6 as a reference. However, the Tmed2 mutant embryos show a severe developmental delay and a smaller size compared to stage matched wild type embryos. As we explain in the text (page 13 line 7) we have selected stages where the neural tube appears of similar morphology in mutants and wild type to facilitate comparison (Suppl. Fig. S6A). These sections are shown in the main figure (Fig. 5A, 5D, and 5E). We have further aimed to control for differences in the developmental timing of Hh signalling. In Tmed2 mutant Hh signalling is delayed (Jerome-Majewska et al. 2010). To further control for potential developmental timing, we have examined markers that are consistent in wild type over the relevant developmental period from E9 to E10.5. The ratio of the ventral marker Olig2 relative to the reference Nkx6.1 (to control for neural tube development) remains nearly unchanged in wild type from E9 to E10.5 (Fig. 5C). In Tmed2 mutants the Olig2/Nkx6.1 ratio is significantly elevated over wild type E9 and E10.5 (Fig. 5C) consistent with a more ventral character of the neural progenitors in the absence of Tmed2. However, we make it clear in the text that the Tmed2 phenotype is not explained alone by misregulated Hh signalling but also involves other defects, in particular development of the posterior trunk of the embryo is severely affected and an overall growth defect is observed (Jerome-Majewska et al. 2010). Our intention for this experiment was to provide evidence in addition to our analysis of in vitro generated neural progenitor cultures. We show that the situation in the embryo is at least consistent with our model. We hope that the reviewer will agree that analysing of the physiologic context does add some value to our study even if the view is much clearer from the cell culture experiments.

14. Page 14, lines 11-13: The authors state that they 'note that the increase in HH signaling in the absence of TMED2 is different from ectopic activation caused by absence of negative modulators'…but don't comment on why this might be the case.
Response: We thank the reviewer for pointing out the missing explanation. We have now included our interpretation in text on page 14 end of first paragraph, and discussion, page 22. From our data it is clear that Tmed2 acts upstream of SMO and regulates its abundance on the plasma membrane. This leads to elevated GPCR and non-canonical outcomes. However, the Tmed2 mutation alone is not sufficient for SMO to enter the ciliary compartment and affect Gli processing. This is regulated by a second step downstream of Tmed2. However, the elevated levels of SMO at the plasma membrane lead to a stronger Gli response upon reception of a Hh signal, thus, a Patched mediated regulatory step downstream of Tmed2 is unaffected in Tmed2 mutant cells. This evidence is consistent over different cell types including 3T3 (SMO is detected at the cilia only after SHH treatment, Fig. 4B), neural progenitors (biochemistry showing SMO at the plasma membrane, Fig. 7G, and quantitation of marker expression, Fig. 4F, and SMO-HA expressing cells in Fig. 7H).

15. Figure 6: For colocalization experiments with ER, Golgi and RAB markers, statistical analysis should be performed. Can the coordinate based colocalization (CBC) test results that are mentioned in line 24, page 15 be subjected to statistical analysis? Quantification provided in 5G is not sufficient - also, need clarification in the text as to what this panel shows. "Quantification showed that 40% of TMED2 is tightly associated with SMO" is not an informative statement. Can Pearson's colocalization coefficients be determined and statistically analyzed?
Response: We thank the reviewer for pointing out the importance to assess colocalization with appropriate statistics. In particular, we have selected Superresolution microscopy as other imaging methods provided insufficient detail for establishing colocalization with high

confidence. Colocalization for superresolution images is more complex as at this resolution chanel shifts and color alignments need to be precisely corrected before measurements can be made. Indeed, subtle shifts in instrumentation are sufficient to introduce considerable artefacts that need to be compensated by appropriate measurements. A framework for colocalization studies has been developed (Malkusch et al. 2012) that we have applied in our study. Briefly, in CBC colocalization between each point form channel A is compared to points in channel B in ray a (100nm) versus ray b where b is larger than a (300nm). The resulting measurement is normalized for volume difference. Each localization of the observed protein population is attributed to an individual colocalization value calculated as Spearman's correlation coefficient between the linearized distribution functions of both protein populations in the neighbourhood of the very localization (Malkusch et al. 2012). We provide additional details in the method section (page 34, line 9) and reference to original work which has developed the approach.

16. Figure 7G: Cell surface biotinylation of SMO is shown in Tmed2 -/- cells compared to WT cells following SHH treatment. Functional experiments (Figure 4A, for example) revealed that Tmed2-/- cells only showed a difference from wild type following SHH treatment. Why are the experimental conditions/conclusions inconsistent?
Response: As explained above we interpret the effect of the Tmed2 mutation as an upstream regulator of SMO. This does not preclude a regulated step downstream of SMO. We believe the transcriptional effect is regulated by a second step that is independent of Tmed2. A mutation of Tmed2 leads to higher levels of SMO at the plasma membrane but does not by itself allow SMO to enter the ciliary compartment or affect Gli processing. The higher abundance of SMO on the plasma membrane affects the strength of the Gli response after a Hh signal is received. We conclude that Tmed2 regulates the GPCR function of SMO and modulates the canonical outcome. We hope that the reviewer will agree that this model is consistent with the experimental observations. (see also response to point 14 above).

17. For changes in SMO molecular weights, the authors speculate that SMO is differentially modified in control vs. Tmed2 null cells, but there is no discussion of why this might be. Have the authors considered that SMO is skipping the Golgi in Tmed2 null cells? This has previously been reported for SMO-M2 (PMC3044124). Or, as the authors propose, it may be due to SMO spending less time in the Golgi in the absence of Tmed2.
Response: We thank the reviewer for brining the study to our attention and raising interesting possibility of a route that skips the Golgi. Our initial idea has been that the higher molecular weight form of SMO might arise from glycosylation in the Golgi and that in the absence of Tmed2 a faster transit might lead to reduced modification and lower apparent molecular weight. We have now additional data and believe that this is an interesting aspect that is presently not completely understood and will need further studies. Briefly, we find predominantly the lower molecular weight form of SMO at the plasma membrane in Tmed2 mutant neural progenitors (Fig. 7G) in the absence of SHH. In contrast, wild type cells show little SMO at the plasma membrane in the absence of SHH, and near exclusively the high molecular weight form of SMO after SHH treatment. However, the situation appears to be different in ESCs. Tmed2 mutant ESCs show the high molecular weight form of SMO at the plasma membrane. It is likely that the molecular weight difference could reflect different traffic routes and/or modification regulation in a cell type specific manner. In particular, the route that potentially bypasses the Golgi (no glycosylation) might not be functional to the same level in ESCs than in NSCs. A full investigation of the posttranslational modification of SMO and its dependence on Tmed2 will need substantial more experiments that we will investigate in the future. Therefore, we have toned down our earlier suggestion that the

modification might indicate SMO localization or be a potential release signal (discussion page 22, last paragraph).

18. The authors looked at two other cell surface proteins (N-cad and EGFR) and saw that their cell surface localization was unaffected by TMED2 loss. From this result, they conclude that TMED2 effects on cell surface localization are 'specific' to SMO. I don't think the conclusion of specificity can be made from looking at only two additional cell surface localized proteins. SMO is probably one of several cell surface-directed proteins that is influenced by TMED2. So, I recommend toning down the specificity argument.
Response: We agree with the reviewer that Tmed2 has been shown to interact with other GPCRs and have cited two relevant studies in our original manuscript (Luo et al. 2007; Stepanchick and Breitwieser 2010). We have analysed other membrane proteins to show that there is no general defect of membrane protein localization in Tmed2 mutant cells. To clarify this we have added a paragraph to the discussion on page 21 (second paragraph).
In the course of our revision we have further obtained new data (addressing point 1 from reviewer 3) that show that Tmed2 also interacts with the closely related GPCR Fzd2 (new Suppl. Fig. S11D). In contrast to SMO, Fzd2 is not restricted by Tmed2 from reaching the plasma membrane. Our data show that Fzd2 at the plasma membrane is unchanged or even slightly reduced in Tmed2 mutant cells (new Suppl. Fig. S11E). These data indicate that the mechanism of Tmed2 in HH signaling is different from other GPCRs and our study shows that specifically SMO is retained by Tmed2 in the ER-Golgi compartment. We have further attempted to obtain antibodies for investigating the interaction of Tmed2 with a range of GPCRs in a more systematic way. Unfortunately, we could not obtain sensitive and specific Western blot detection with any of these commercial antisera in the time frame of this revision.

19. The authors speculate that release of SMO from the Golgi is a key event that is stimulated by HH pathway induction. This hypothesis stems from their observation that the majority of their SMO signal is evident in the ER and Golgi compartments. However, the localization studies are conducted using over-expressed SMO, and it's unclear whether these conclusions would apply to protein expressed at endogenous levels. Over-expressed cell surface proteins typically pile up along the secretory pathway, and may not represent the trafficking behavior of a protein expressed at physiological concentration. Thus, I am not persuaded by the argument.
Response: The reviewer raises an important point and we agree with the reviewer that knowledge of the intracellular localization of endogenous SMO is important. We have not been successful in detecting endogenous SMO expression by antibody staining except when it is localized at the primary cilia. Examining the literature, this seems to be the case in a large number of studies. In contrast, our HA-tagged SMO expression construct can be sensitively detected, when it is expressed at 5 to 10 times the endogenous level. This has motivated us to attempt to tag the endogenous SMO gene with a 3xHA tag using a CRISPR induced oligonucleotide mediated repair strategy (new Suppl. Fig. S8). We show now in new Fig. 6I and Suppl Fig. S8D and S8E of our revised manuscript images of the tagged SMO protein at endogenous level. The signal of endogenous HA-SMO appears to be extremely faint and we were only successful in imaging after selecting a sensitive HA antiserum. Our analysis has already revealed two important new observations (page 15, second paragraph). Firstly, the localization of SMO at the endogenous level resembles closely the distribution we have observed with our SMO-HA expression construct. Secondly, we find that a population of SMO protein colocalizes with Tmed2 (Fig. 6I). Thirdly, we observe heterogeneity of SMO expression among cells likely indicating that Smo expression is regulated. Taken together our

data strongly suggest that results obtained with our SMO-HA expression construct are relevant for the endogenous protein. At the moment our sensitivity for imaging SMO at the endogenous level is limited and unfortunately not high enough for obtaining super-resolution images. More work will be required for signal amplification and to account for expression heterogeneity. The cell lines generated in our study will provide a basis to further study SMO localization in the future.

Minor point: Page 4 lines 11-13 of introduction: The authors state the nature of the SHH response (canonical or noncanonical) is determined prior to SMO cycling through the primary cilium. However, there may be biased agonists that control the SMO effector route selection, which would represent a trafficking independent route selection process. The authors should also consider that SMO glycosylation may impact the GLI vs. non-GLI response (PMC4546403).
Response: We thank the reviewer for the comment and pointing out the literature reference. We appreciate that correlation of SMO glycosylation, trafficking and signalling outcome are complex. We have therefore reconsidered our original view and have rephrased the revised text adding the possibility of alternate route selection in the discussion on page 22 (also *see* response to point 17).

Response to the comments raised by reviewer 2

Rev. 2:
Minin and colleagues present some novel data on a potential role of TMED2 in the regulation of Hedgehog signaling through limiting SMO trafficking to plasma membrane. The authors firstly demonstrated that SMO is critical for ESCs survival and this function is independent of transcription factor GLIs. Based on the finding that SMO GPCR function is important for ESC survival, they then identified TMED2 mutant enhances SMO GPCR function and elucidated its mechanism. The concept for this paper is novel and the conclusion is supported by their data. There are still some concerns regarding the manuscript.
Response: We thank the reviewer for finding our study of interest and the helpful comments.

1) Fig.2 and Fig. S2 provide much information in the effect of PMP/SAG on GLI activation and SMO-mediated ESC survival. Several confusing points in Fig. 2 and Fig. S2 need to be addressed. In Fig. S2F, two independent SMO-/- clones showed different results upon high dose PMP / SMO treatment. Statistical analysis is needed here, as this result is critical for understanding whether PMP works at the upstream of SMO or not.
Response: We have included a T-test in Suppl. Fig. S2F following the reviewer's request. The differences between the two SMO -/- clones do not reach statistical significance.

In Fig S2H and Fig S2I, it is interesting that ROCK inhibition and Rac1 (CA) overexpression can only rescue half (but not all) cell death led by PMP treatment. An explanation for it may be needed
Response: We observe that treatment with ROCK inhibitor and overexpression of Rac1(CA) affects cell viability in the absence of Purmorphamine (Suppl. Fig. S2H and S2J (new) comparison between black bars). Therefore, we attribute the incomplete rescue as a result of adverse effects of the inhibitors. We would like to point out although the rescue appears incomplete it is nonetheless significant. The positive effect on cell viability contrasts the negative effect of the treatment in the absence of PMP, which could be considered a baseline for comparison.

In Fig 2C and 2D, why does total amount of SMO-HA decrease dramatically by treatment of SAG or PMP, especially the lower band in PMP-treated sample in Fig 2D?
Response: We would like to respond to the reviewer's point in the following way. It has been established that high concentration of PMP and SAG have an inhibitory effect on SMO but the mechanism of this inhibition is presently not known. Our analysis by Western blot would suggest that loss of SMO protein likely by turnover or destabilization is correlated with PMP mediated inhibition. A shift between the two molecular weight forms would be consistent with enhanced turnover. However, we also consider now that route selection might affect the apparent molecular weight of SMO, which we would like to investigate in a future study.

2) In page 9 line 23,"Treatment of ESCs with high PMP concentrations allows to quickly induce SMO destabilization", more data is needed to demonstrate that the decreased amount of SMO-HA by PMP treatment is due to PMP-induced SMO destabilization, but not other reason.
Response: We agree with the reviewer that we did not show destabilization as the cause of reduced SMO protein levels. We have rephrased the text and now avoid stating a specific mode of loss of SMO protein. We consider turn over as a likely explanation. Conceivably, also translation or transcription of SMO could be affected by PMP but we consider this less likely based on the evidence that PMP can bind SMO protein.

3) The fluorescent staining method for SMO-HA in Fig. 2C&3E, as well as ERP72, RCAS1 and RAB7 in Fig.3C need to be described.
Response: We thank the reviewer for pointing out the missing detail. We now specify the detection method in the figure legends (Fig. 3C to 3E).

4) In Fig. 3E, the authors showed the immunostaining result of one representative cell. However, a quantitative or semi-quantitative experiment need to be performed as the results from cell population are more credible. Western Blot is a good choice to check SMO changes in separated components of cell lysate, such as plasma membrane, cytosol and nucleus.
Response: We agree that immunofluorescence is not suitable for quantifying the localization of SMO. We have exchanged this experiment with a new Western analysis of SMO in Tmed2 -/- cells. New Fig. 3G shows a reduction on SMO protein (particularly the higher molecular weight form) after PMP treatment in wild type and to a lesser extent in Tmed2 mutant cells. In contrast to NSCs we do observe also the high molecular weight form of SMO in Tmed2 -/- ESCs. The apparent cell type difference in SMO glycosylation might be explained by the availability of a Golgi independent route of SMO in NSCs that is not functional in ESCs. However, we will need more time to perform a comprehensive analysis and would like to address this aspect in a future study.

5) In Page 11 line 22-23, "PMP also decreased SMO levels in Tmed2 mutant ESCs but not to the extent as in control cells", the related result of Western blot should be shown.
Response: We have included the Western analysis in new Fig. 3G as requested by the reviewer (see point 4 above).

6) In Fig. 6, why are the shape of cells and the expression pattern of SMO different with those shown in Fig.2c & 3c?
Response: The samples represent different cell types and were prepared differently. For imaging ESCs we have prepared a single cell suspension and used cytospin. Neural progenitors were directly fixed on slides with paraformaldehyde.

Although the scale bar 5 mm has been marked, the cells in 6C and 6E look much bigger than those in Fig. 6B and 6D. Please check the original data.
Response: The scale bars in the panels A to D and panel E in figure 6 is of different length and the size is written in the figure panels above the scale bar. We have checked the scale bar length and it correctly represents the image magnification.

Overall this is a good quality study and should be of interest to the audience.
Response: We thank the reviewer for the detailed comments that have helped to improve our study and the positive assessment.

Response to the comments raised by reviewer 3

Rev. 3:
In this manuscript Di Minin et al. investigate a novel interaction between Tmed2/10 and the essential Hh-pathway component Smoothened. They show that non-canonical Smo function is required for the survival of ES cells and that very high levels of the Hh pathway agonists SAG or Purmorphamine inhibit Smo activity and decrease ES cells survival similar to loss of Smo. Based on these observations, they perform a CRISPR-screen in haploid ES cells to identify novel genes that confer resistance to treatments with high levels of Purmorphamine, which identifies Tmed2/10 as candidate genes. The authors confirm a role of Tmed2 in the Hh pathway by characterizing gene expression patterns in neural differentiations and the in-vivo spinal cord, super-resolution imaging and extensive biochemical analysis. They propose a model in which Tmed2 retains Smo in the ER/Golgi in the absence of Hh pathway activation. This interaction is quickly released upon Hh pathway activation. As this release occurs before other well-known steps of Hh pathway activation, e.g. accumulation of Smo in the primary cilium, the authors conclude that the release of the Tmed2/Smo interaction is one of the earliest and most-upstream interactions in the Hh pathway known to date. Overall this is a strong manuscript that uses state-of-the-art techniques to address an interesting problem, the upstream regulation of the Hh pathway. The experiments are thoroughly conducted and the results are consistent. I therefore believe that this manuscript will be of interest to a wide-audience. I have couple of comments/suggestions, which I feel the authors need to address through additional experiments prior to publication, but I believe that they will be able to do so during a normal revision.
Response: We thank the reviewer for the positive comments.

Major comments
1. Specificity of the Tmed2/Smo interaction. The authors try to show at multiple places in the manuscript that the phenotypes they observe are specific due to the interaction between Tmed2 and Smo. Yet, the phenotypes of the Tmed2 null embryos are different from those typically observed in mutants that disrupt the Hh pathway, suggesting that Tmed2 may also play a role in other cellular processes or affect other signalling pathways. This concern is further exacerbated by the fact that the actual patterning phenotype observed in the neural tube is comparably mild, e.g. the expression domain of the low-threshold target gene Nkx6.1 does not appear to be strongly expanded dorsally and the ventral target gene Olig2 is also expressed, suggesting that the Hh pathway is only mildly disrupted in these mutants. I therefore think the authors need to check if the Tmed2 mutation also affects the activity of other signalling pathways that control patterning of the spinal cord or survival of ES cells. Of specific concern here is the Wnt pathway as it relies on GPCRs closely related to Smo for its activity.
Response: We thank the reviewer for bringing up this important point. We have performed additional experiments to investigate if Tmed2 and the SMO related GPCR Wnt receptor Fzd2 can interact. Suppl. Fig S11D shows new data of a CoIP experiment in 293T cells indicating that Fzd2 can bind TMED2 similarly than SMO. This observation is consistent with other studies showing interactions between GPCRs and Tmed2, which we have cited in our original submission (Luo et al. 2007; Stepanchick and Breitwieser 2010). We further investigated the effect of Tmed2 on plasma membrane localization of Fzd2. New Supp. Fig. S11E shows biochemical purification of plasma membrane proteins in WT and Tmed2-/- cells expressing HA-Fzd2. Unlike SMO, Fzd2 is not restricted by Tmed2 (page 18, end of last paragraph). Our experiment shows that there is less Fzd2 at the plasma membrane in the Tmed2 mutant cells than in wild type. However, we also identify a role for Tmed2 in Fzd2

glycosylation (the higher molecular weight band of Fzd2 is weaker in the input in Tmed2 -/- cells). Therefore, we conclude that Tmed2 can interact with Fzd2 but it does not restrict its trafficking to the plasma membrane. Tmed2 is likely to also play a role in Fzd2 trafficking which is beyond our present study. Our in vitro assays (ESCs and NSCs) assess HH dependent effects and we can show the effects of Tmed2 depletion on SMO trafficking. In vivo, we cannot fully exclude the role of additional signaling in the Tmed2-/- phenotype. From earlier work it is reasonable to assume that not all aspects of the mouse phenotype are explained by effects on Hh signaling (Jerome-Majewska et al. 2010) and likely other pathways are also affected in Tmed2 mutant mice. Considering the prominent role of Hh signaling in neural tube patterning our observations in vivo appear at least consistent with a misregulation of Hh signaling in Tmed2 mutant embryos. We believe that analyzing the phenotype in the physiological context adds to our biochemical analysis and cell culture experiments, which provide more possibilities to rule out other causes. We have added a statement to the revised text (discussion page 21, second paragraph) that we do not rule out that additional effects of Tmed2 on other pathways could contribute to the phenotype.

Minor comments

1. The authors claim that non-canonical Smo signalling is required for ES cell survival based on their observation that ROCK inhibitors or expression of a dominant-active Rac1 can rescue loss of Smo or Gi inhibition by Pertussis toxin. However, these treatments may just generally improve cell health or inhibit apoptosis independent of Smo activity. Do the authors observe any changes in the expression levels or activity patterns of endogenous Rock1 or Rac in Smo mutant ES cells or upon treatment with high levels of Purmorphamine?
Response: For answering this important question we have performed additional experiments. Firstly, we tried to analyze the ratio of Rac1 GTP to GDP using a biochemical pulldown assay based on a GST fusion of the p21 binding domain of p21-activated kinase, which has affinity for Rac1-GTP. However, our experiments had substantial variability and we were unable to find conditions that gave reproducible results leading us to give up on this approach in the end.
Next we aimed to assess if myosin contractility would be elevated after PMP treatment. For this we analyzed MLC2 phosphorylation (non-muscle myosin light chain 2, a known substrate of ROCK) by immunofluorescence staining. We detect intense cortical MLC2 phosphorylation in wild type ESCs after PMP treatment consistent with elevated cortical contractility as a prominent downstream effect. In contrast, Tmed2 mutant ESCs did not show elevated MLC2 phosphorylation in the presence of PMP and the staining was homogenous and comparable to untreated wild type cells (new Suppl. Fig. S2I). These data further support our earlier conclusion that myosin contractility is activated in ESCs after PMP treatment and plays a role in mediating the cell death response.

2. The authors show that canonical signalling via Gli transcriptional activity does not work in pluripotent cells. Previous work suggested that under 2i+LIF culture conditions, similar to those used by the authors, only a small percentage of cells has primary cilia (Bangs et al. Nat. Cell Biol. 2015). I therefore wonder if there is any correlation between activation of the Gli-dependent branch of Hh signalling and the presence of primary cilia in ES cells. The authors should at least check what percentage of ES cells in their cultures exhibit primary cilia.
Response: We agree with the reviewer that absence of a primary cilia could be a likely reason for the unresponsiveness of Gli activity in ESCs. We have performed immunostaining and counted the cells with a clearly recognisable cilia. We find that 20% of ESCs do show a primary cilia (new Suppl. Fig. S1H), which is consistent with the earlier report. We thank the reviewer for pointing out the earlier study, which we now cite in the text on page 6, line 24.

3. The Tmed2 rescue of high purmorphamine levels correlated with an increase of Smo at the plasma membrane. The authors argue later in the text that higher levels of Smo at the plasma membrane in Tmed2 mutants make cells hypersensitive to Shh and that treatments of NPCs with low levels of purmorphamine are sufficient for Smo relocalization from internal to external cell compartments. This leads to the questions if 1) SAG, Purmorphamine or Shh treatments cause a similar relocalization of Smo in ES cells, and 2) if the interaction between Tmed2 and Smo in ES cells is as quickly disrupted in response to Hh pathway activating compounds as the authors show later in the paper for NPCs? Addressing these questions would allow the authors to further pinpoint which aspects of the Hh pathway (upstream or downstream of Tmed2) are disrupted in ES cells.

Response: We have performed additional experiments to address the reviewer's question. We include the new data analysing SMO at the plasma membrane in ESCs treated with SHH and the low (activating) concentration of PMP in this response for the reviewer. In contrast to neural stem cells we do observe only a small effect on the abundance of SMO at the plasma membrane (panel A below). Therefore, it appears that neither SHH nor PMP can relocalize SMO in ESCs. In addition, we have investigated the interaction between SMO and Tmed2 in ESCs (panel B below). Consistent with the unchanged membrane localization, neither SHH nor PMP seem to affect the interaction between SMO and Tmed2 in ESCs. We interpret these new observations in the following way. Differently to NSCs (and 3T3) the abundance of SMO at the PM in ESCs seems to be quite high in the absence of Hh ligands or PMP, and does not measurably increase upon SHH and PMP treatment. Indeed we do observe a small decrease but this is likely due to a technical issue (panel A below). In addition, SMO binding to TMED2 also remains unchanged by SHH and PMP. Therefore, there is a substantial difference between ESCs and NSCs. A potential explanation could be that Ptch1 is not active and ESCs do not respond to SHH and a low dose of PMP might not reach the ER resident pool of SMO. Conversely, SMO appears not strictly retained in the intracellular compartment in ESCs as it appears to be in NSCs leading to a higher amount of SMO at the PM. The higher SMO pool might be needed to prevent cell death. The absence of a Gli response in ESCs might be explained by a second regulated step and possibly by the absence of a clearly defined primary cilia in the majority of ESCs. From our investigation of Hedgehog signalling we also have obtained preliminary evidence that neither mutation of Ptch1 nor expression of an activating mutant SMO-A1 are sufficient to induce GLI activity in ESCs. In contrast, a Sufu mutation, which we would place downstream of the cilia, increases Gli activity modestly. From these observations we speculate that the upstream part - potentially including the ciliary compartment - might be missing or be substantially different in ESCs. Taken together our observations suggest that regulation of SMO in ESCs is complex and future work will be needed to address the details. Investigation of trafficking routes of SMO (also see response to point 17 and minor point raised by reviewer 1) using ESCs with the endogenous tagged SMO (also see response to point 19 raised by reviewer 1) will provide an opportunity to study these aspects in the future.

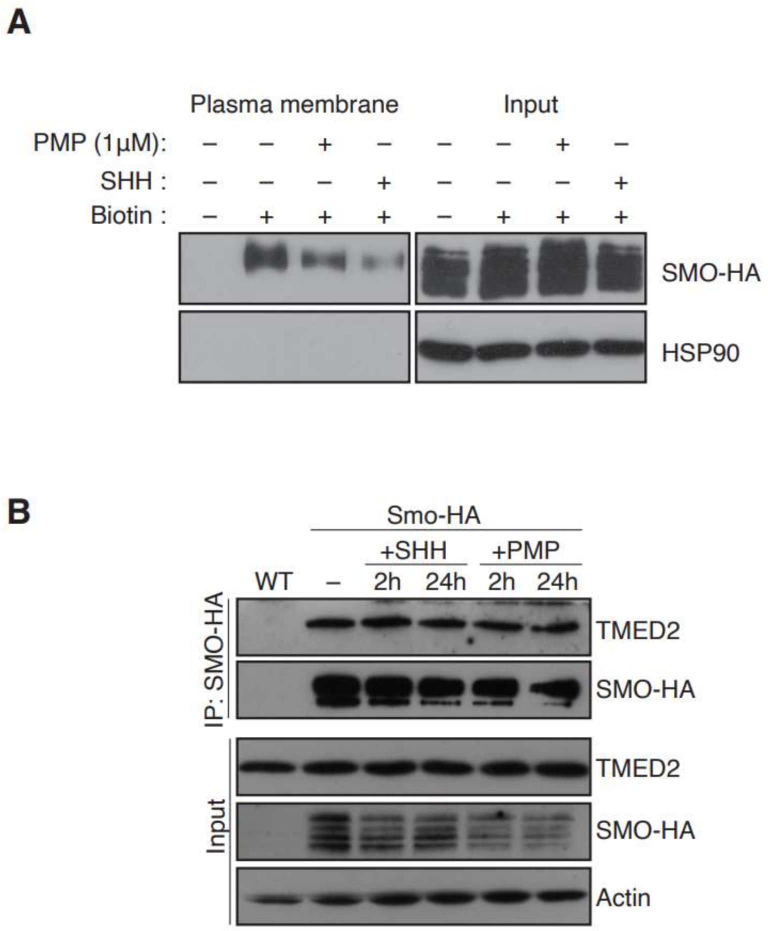

4. Pg 13 lines 9-11: The authors say they compared the expression of Nkx2.2 and Olig2, but as far as I have seen they only show data for Olig2. They should add the data about Nkx2.2 as it might provide a more sensitive read-out due to its more ventral expression

Also, levels and duration of Shh signalling are linked in the ventral spinal cord (e.g. Dessaud et al. 2007, Balaskas et al. 2012) and the authors mention a developmental delay in the Tmed2 mutants. The authors should perform a more detailed developmental time-course of Shh target gene induction in the Tmed2 mutant mouse embryos to ensure that their e10.5 Tmed2-mutants are exposed to Shh for similar durations as their e9.0 controls

Response: We have shown the Nkx2.2 staining in Fig. S6A. To make this more readily accessible we have added the marker description in the figure panel.

We agree with the reviewer that the timing of SHH signaling in development is an important aspect for ventral marker expression. In a previous study the expression of Shh has been studied in E8.5 Tmed2 mutant embryos and shown to be delayed compared to control embryos (Jerome-Majewska et al. 2010). Shh expression in mutant E8.5 embryos is comparable to E7.5 wild type embryos. Therefore, we believe our comparison of E10.5 Tmed2 mutant to E9 wild type neural tubes is not completely asynchronous. We have been looking for developmental markers that would not be affected over time in wild type and identified that the ventral marker Olig2 and the reference Nkx6.1 (to control for overall neural tube development) show a relatively constant ratio from E9 to E10.5 (Fig. 5C). We find that the ratio of the ventral marker Olig2 to Nkx6.1 is increased in Tmed2 mutant neural tubes, which would be expected if the patterning is ventralized. However, we do acknowledge that an interpretation is difficult. We believe that analysing the phenotype in the physiological context add to our results in cell culture, where other effects can be controlled much more convincingly. Our results in vivo appear to be at least consistent with a

ventralization of neural tube patterning in Tmed2 mutant embryos.

5. Pg 14 lines 7-9: The authors only look at one marker (Pax7) here. They should add additional markers to ensure this effect is not Pax7-specific. Also, the dorsal boundary of Nkx6.1 does not seem strongly shifted in the Tmed2 mutants, suggesting that the intermediate Dbx1/2-positive domain may be strongly expanded. Is this the case?
Response: We thank the reviewer for the suggestion to investigate Dbx1. We have now performed staining of neural tube sections using a Dbx1 antibody (kindly provided by the Pierani lab). These new experiments show a dorsal expansion of the Dbx1 domain as the reviewer had suspected (new Fig. 5D, text page 14, first paragraph). Taken all marker analysis together a consistent view emerged. We believe that Tmed2-/- neural tube resemble the description of the Gli3-/- mutant (Persson et al. 2002) to a large extent. Both the Tmed2 and Gli3 mutations show less pronounced changes in the ventral region compared to Ptch1 mutant. We see a small expansion of Nkx6.1, an expansion of Dbx1 domains, and repression of the Pax7 domain. The Tmed2 mutation increases the amount of SMO that responds to Hh signals but there is no ectopic Gli activation as is observed with the Ptch1 mutation. Nevertheless, the increased abundance of SMO on the plasma membrane in Tmed2 mutant neural tubes is sufficient to modulate HH signaling output similar to a loss of GLI3 repressor function.

6. Pg 18 lines 15-18: The authors look at 2 proteins (EGFR and N-Cadherin) with very different structures to Smo to show that the Tmed2 mutation has a specific effect on Smo trafficking. I would find this argument more convincing if the authors would have also looked at proteins with more similar structures, e.g. other GPCRs, to ensure that their trafficking is not perturbed.
Response: We agree with the reviewer that Tmed2 can interact with other GPCRs and have cited two studies in our original submission (Luo, et al., 2007 and Stepanchick et al., 2010). To further address this point we have performed additional experiments to investigate if Tmed2 and the SMO related GPCR and Wnt receptor Fzd2 can interact. A CoIP experiment in 293T cells indicates that Fzd2 can bind TMED2 similarly to SMO (new Suppl. Fig. S11D). We further investigated the effect of Tmed2 on plasma membrane localization of Fzd2. Biochemical purification of plasma membrane proteins in WT and Tmed2-/- cells expressing HA-Fzd2 indicate that Fzd2 is not restricted by Tmed2 (new Suppl. Fig. S11E). The experiment might suggest a role for Tmed2 in transport of Fzd2 for reaching the plasma membrane and in glycosylation. Therefore, the mechanism of restriction of SMO is not observed with Fzd2. It is likely that Tmed2 also plays a role in Fzd2 trafficking which is beyond our present study. Our in vitro assays (ESCs and NSCs) assess HH dependent effects and we clearly show the effects of Tmed2 depletion on SMO regulation, trafficking. In vivo we cannot fully exclude the role of additional signaling in Tmed2-/- phenotype. From earlier work it is reasonable to assume that not all aspects of the mouse phenotype are explained by effects on Hh signaling (Jerome-Majewska et al. 2010) and likely other pathways are also affected in Tmed2 mutant mice. Considering the prominent role of Hh signaling in neural tube patterning our observations in vivo appear at least consistent with a misregulation of Hh signaling in Tmed2 mutant embryos. We believe that analyzing the phenotype in the physiological context adds to our biochemical analysis and cell culture experiments, which provide more possibilities to rule out other causes. We have added a statement to the revised text that we do not rule out that additional effects of Tmed2 on other pathways could contribute to the phenotype.

7. Pg 19, lines 9-11: The authors state that some Tmed2 phenotypes are due to the

combination with the Smo-HA transgene. For better comparison, the authors should add levels of the corresponding target genes in Smo-HA controls without Tmed2 mutation and Tmed2-mutants without Smo-HA transgene to the corresponding plots in Figure 7 and S10.
Response: We have performed a new analysis of Olig2 and Nkx2.2 expression in neuralized EBs from wild type, SMO-HA expressing, and SMO-HA Tmed2 mutant ESCs (new Fig. 7H). These data show that an upregulation of ventral markers in the absence of exogenous SHH is not observed and the cells with or without the Tmed2 mutation and SMO-HA expression construct behave similar in the absence of SHH. Upon addition of SHH we observe a progressively increased number of cells expressing ventral markers from wild type to SMO-HA expressing cells, to the highest amount in Tmed2 mutant SMO-HA expressing cells. These results are consistent with the SMO levels at the plasma membrane controlling the magnitude of the response. We have previously shown on the RNA level an increase of ventral marker gene expression in SMO-HA expressing Tmed2 mutant cells in the absence of SHH. We note that this increase on the RNA level does apparently not lead to a detectable increase on the proteins level and was modest. Our new data with ESCs expressing an engineered 3xHA tagged version of the endogenous SMO gene (see response to point 19 from reviewer 1) suggest an explanation for our earlier observation. We observe that expression of endogenous SMO is heterogeneous with about 30 percent of the cells showing a clear sign of expression in immunofluorescence staining. Introduction of our expression vector therefore will lead to an increased number of SMO expressing cells that can then result in a corresponding increase of cells with ventral marker expression. This interpretation is consistent with our observation that neither heterologous (over) expression of SMO-HA nor the Tmed2 mutation does ectopically activate Gli activity in the absence of HH ligands. We had suspected that a combination of SMO overexpression and Tmed2 mutation might have had a synergistic effect, but consider this now unlikely based on our new experiment in Fig. 7H. The 5 to 10 fold higher expression of SMO from our expression vector appears to resemble the localization and function of endogenous SMO closely, with the exception that endogenous SMO expression is restricted to fewer cells.

Typos and small corrections

Figure S1C: Remove "Figure 4" text box
Response: We have removed the text box.

Pg 6 line 4: The authors should clearly state if they are looking at mouse or human ES cells. This should also be mentioned in the Experimental Procedures section.
Response: We thank the reviewer for pointing out the missing detail. The revised text clearly states that mouse ES cells were analysed (Abstract, line 4, and section heading page 9, last paragraph).

Pg 21 line 9: NCPs should be NPCs
Response: We have corrected the mistake.

References

Jerome-Majewska LA, Achkar T, Luo L, Lupu F, Lacy E. 2010. The trafficking protein Tmed2/p24beta(1) is required for morphogenesis of the mouse embryo and placenta. *Dev Biol* **341**: 154-166.

Luo W, Wang Y, Reiser G. 2007. p24A, a type I transmembrane protein, controls ARF1-dependent resensitization of protease-activated receptor-2 by influence on receptor trafficking. *J Biol Chem* **282**: 30246-30255.

Malkusch S, Endesfelder U, Mondry J, Gelleri M, Verveer PJ, Heilemann M. 2012. Coordinate-based colocalization analysis of single-molecule localization microscopy data. *Histochem Cell Biol* **137**: 1-10.

Persson M, Stamataki D, te Welscher P, Andersson E, Bose J, Ruther U, Ericson J, Briscoe J. 2002. Dorsal-ventral patterning of the spinal cord requires Gli3 transcriptional repressor activity. *Genes Dev* **16**: 2865-2878.

Stepanchick A, Breitwieser GE. 2010. The cargo receptor p24A facilitates calcium sensing receptor maturation and stabilization in the early secretory pathway. *Biochem Biophys Res Commun* **395**: 136-140.
