# Supplementary material for: TMED2 binding restricts SMO to the ER and Golgi compartments
Source: PLoS Biol. 2022 Mar 30;20(3):e3001596. doi: 10.1371/journal.pbio.3001596 (PMC9000059; doi:10.1371/journal.pbio.3001596)
Supplement: Attachment — Submitted filename: point_to_point_response.pdf [file pbio.3001596.s019.pdf]

Response to the Reviewers' comments

Response to Reviewer 1

The revised submission is improved from the initial manuscript draft. However, some of the results remain over-interpreted, and speculation is presented as a solid conclusion on more than one occasion. The authors state that the SMO signal they investigate in ESCs is non canonical GPCR signaling. They did not conclusively show this, so the more conservative way of referring to what they are looking at is 'GLI-independent' SMO signaling since ESC viability appears to be insensitive to GANT. The PTX result +/- SMO suggests a SMO-Gi signal, but does not specifically show it is responsible for the observed effects. There is increasing evidence for a ciliary GPCR network that impacts SHH signaling, so assuming SMO is the relevant GPCR without directly showing SMO-Gi activation is risky.

Response: We thank the reviewer for the specific comments. We agree with the reviewer that the term non-canonical is unnecessary and prone to misinterpretation from its definition as an exclusion. We now use the more specific term Gli-independent (page 7, last paragraph, and section heading on page 6). We also have considered the reviewers view that we cannot be absolutely certain that the relevant GPCR is SMO alone or that the G-protein mediated response is entirely due to Gi activation. Although, it is clear that all our results are consistent with SMO Gi activity, it would be impossible to rule out contributions of other G-proteins and less likely other GPCRs (these would also need to be affected by both SAG and Purmorphamine). Our conclusion on page 6, first paragraph is carefully worded to avoid overstating the evidence. However, we have changed the section heading on page 6 to now not specifically implicate SMO GPCR activity.

The authors do show that Tmed2 is involved in SMO ER-Golgi-Plasma membrane trafficking, but I'm not convinced that the increase in plasma membrane SMO that they see in response to SHH is due to specific control of Tmed effects on SMO as is suggested in the manuscript. This is speculation.

Response: Our argument for the involvement of Tmed2 in regulated activation of HH signaling comes from the observation that the biochemical interaction between Tmed2 and Smo is sensitive to HH signaling. Our observation of the dissociation of SMO from TMED2 within one hour further is consistent with a direct effect. However, we agree with the reviewer that alternative explanations cannot be fully ruled out at the time. We acknowledge that a HH signal through PTCH1 might prevent SMO trafficking through the ER-Golgi route and therefore the interaction of SMO with TMED2 would not be abrogated but not occur in the first place. We point this possibility out in the discussion (page 22, end of first paragraph). However, this would not solve the problem that an intracellular regulation of SMO exists that determines its trafficking route from the ER to the Golgi. Considering that Tmed2 is in a central position, this route would need to be discovered by dynamics studies.

The authors did not directly address the question from the initial review of whether cyclopamine might be nonspecifically affecting cell viability. They explained that they are using KAAD-cyclopamine, which is not what was asked.

Response: We thank the reviewer for the comment. We were unable to identify any literature reference that would indicate a different mode of action between Cyclopamine and KAAD-cyclopamine. KAAD-cyclopamine is a derivative and generally preferred due to its pharmacokinetics. We have now performed the experiment using Cyclopamine and observe identical results that no cell death is induced (see figure below for the reviewer). Since there is no new observation we did not include this into the main figure, which shows KAAD-Cyclopamine and SAG.

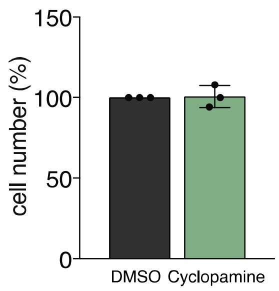

Page 5, line 4 - reference for SMOA1 trafficking is needed.

Response: We thank the reviewer for pointing out the missing citations of reference (REF [38]).

I wanted the authors to add GANT to the panel shown in Sup Fig 1D to test for changes in Gli1 expression in the ESCs. Instead, they checked for effects on ESC viability - not the same thing as gene expression. Since they didn't show the experiment that was specifically requested, I'm left with the feeling that the result of GANT treatment on Gli1 expression was not consistent with the model the authors are trying to promote.

Response: We agree that we have misunderstood the reviewer's request and have added new data showing the transcriptional effect of GANT61 on Gli1 in Fig. S1F. We observe a decrease in Gli1 expression, which contrasts the effect of SAG and KAAD-cyclopamine that did not elicit a transcriptional change. We explain this that GANT61 directly acts on GLI factors, whereas SAG and KAAD-cyclopamine affect SMO function. The lack of a HH response is likely linked to the absence of cilia in ESCs, and therefore lack of GLI processing. Mammalian GLI1 is already in its activator form and expressed in ESCs. This suggests that the Gli transcriptional activity observed in ESCs stems from GLI1, which is reduced by GANT61 but not SAG or KAAD-cyclopamine. We hope that our explanation can convince the reviewer that our data are consistent with our model.

Figure 1H - is Ptch expressed in the ESCs? If so, SMO will not be active without pathway induction, so there is no reason to believe it would be tonically activating Gi.

Response: We thank the reviewer for the comment. Indeed, we detect Ptch1 mRNA in mouse ESCs. ESCs also express DHH, which indicates a potential autocrine signaling loop. Together with the measured Gli activity these data would indicate that SMO is active in mouse ESCs. In addition, we have generated Ptch1-/- ESCs and from our preliminary characterization these do not show a proliferation defect. Taken together these data would indicate that SMO activity is present in ESCs and several components of the HH signaling pathway are present in ESCs.

Page 9, line 1 - it would be more conservative to write: "To investigate how GLI-independent SMO signaling activity is inhibited by high concentrations of PMP..." instead of specifying SMO GPCR signaling. You have not proved it is GPCR activity. The PTX effects are correlative, and do not conclusively prove that you have a Gi signal occurring downstream of SMO in the ESC system.

Response: We have followed the reviewers comment and changed the phrasing as suggested (page 7, last paragraph).

Figure 2E - Does over-expressed SMO accumulate on the membrane as the glycosylated form of the lower-molecular weight form?

Response: We observe that both the high and low molecular weight forms of SMO are detected in ESCs. We now provide a new Western blot in revised Figure S2L showing the two bands of SMO also

in the SMO-HA overexpressing wild-type ESCs. These ESCs express our HA-tagged Smo transgene in addition to endogenous Smo.

The addition of the neutralized bodies is nice and illustrates the hyperresponsiveness to SHH of the Tmed2-/- cells. Because this result illustrates the point, I do not think it is necessary to show the neural tubes that are not age matched in the supplement. Interpretation of the data from non-age matched neural tubes is too speculative.

Response: We thank the reviewer for appreciating our experiment in neuralized EBs. We also agree with the reviewer that our analysis in the embryo is difficult due to the developmental delay of the Tmed2 mutation. However, we are confident that two aspects of the neural tube sections will be of high interest for the scientific community. Firstly, we provide a reference for neural tube markers in Tmed2 mutant embryos, which has not been analyzed before. Secondly, from our extended analysis we feel confident to make specific observations of patterning changes that are unlikely explained by a developmental delay alone. In Fig 5 we provide data on relative expression of a combination of markers that is independent of the developmental timing in wild type mice. Secondly, our observation that Nkx6.1 and Dbx1 domains are juxtaposed in Tmed2 mutant but clearly separated by a wide gap in wild type neural tubes adds to our interpretation of a patterning change. We do acknowledge that the developmental phenotype of the Tmed2 mutation is severe at this time point and much of it is not explained by HH signaling. Importantly, the HH-related component of the phenotype in the neural tube resembles that one of the Gli3 mutation. We think that this observation will be of high interest to readers interested in neural development. We also have toned down and now refrain from concluding that we are certain that the patterning changes are specifically caused by elevated HH responsiveness (discussion page 20, end of first paragraph). However, we still consider this very likely based on our finding in neuralized EBs. We do not want to rule out that other signaling pathways might also be affected and contribute to the patterning changes in Tmed2 mutant embryos.

I'm not sure 'ciliar' is a word - 'ciliary' is clearer.

Response: We have changed the spelling following the reviewer's suggestion (page 16, start of the first paragraph).

Response to reviewer 2:

All previous concerns have been addressed in the revision.

Response: We thank the reviewer for the positive assessment of our revision.

Response to reviewer 3:

The authors have addressed all my points raised on the previous version of the manuscript through additional experiments and by making changes to the text.

Response: We thank the reviewer for the specific comments and the positive evaluation of our study.

My remaining comments are minor. These are:

1.) Pg. 13, L12-14: "Tmed2-/- embryos showed a split OLIG2 domain that was shifted dorsally relative to E9.0 controls, which showed a single ventral OLIG2 domain (Supplemental Fig. S6A)." I am not entirely sure what the authors mean here with split Olig2 domain. Split by the lumen of the spinal cord into the left and right side? Comparing the first 2 embryos in Figure S6A (Tmed2- at e10,5 and WT at e9.0), these look pretty similar. Also the mentioned dorsal shift of the Olig2 domain in the mutants is not very apparent. So I feel the authors need to change the statement or need to at least better support it by a statistical quantification. Also given that the authors compare embryos from different stages of development here, it is unclear to which extent small differences in the position of DV domains are meaningful.

Response: We thank the reviewer for the comment. Indeed, we did observe a clear split of the OLIG2 domains in the mutants as they are shifted to a higher (dorsal) position. However, we realize that our description of the pattern is confusing as also in wild type sections the OLIG2 domain is not at the floor plate and, hence, actually also split - albeit in a very subtle manner. We have eliminated the description of the "split" pattern and refer now only to our evaluation of developmental markers (page 12, first paragraph), which we think best represents a patterning shift in a manner that is highly likely not due to a developmental delay or developmental timing mismatches.

2.) Pg. 14, L2-3: "Consistent with this we also observed an expansion of the intermediate DBX1 expression domain in Tmed2 mutants (Fig. 5D, and Supplemental Fig. S6D).". While there seems to be a small expansion of the DBX1 domain in Fig. 5D, this is much less clear in the embryos shown in Figure S6D. Again I feel that this point should be better supported by some kind of statistical analysis.

Response: We have further analyzed the marker expression in the neural tubes and added statistics (new Fig S6E). We show changes in patterning of Dbx1, whereby the domains of DBX1 and NKX6.1 are juxtaposed in Tmed2 mutant but clearly separated by a wide gap in wild type neural tubes. This observation has been reproduced in 3 WT and mutant embryos. We point this now out on page 12, end of first paragraph.
